# C-type lectin 4 regulates broad-spectrum melanization-based refractoriness to malaria parasites

**DOI:** 10.1371/journal.pbio.3001515

**Published:** 2022-01-13

**Authors:** Maria L. Simões, Yuemei Dong, Godfree Mlambo, George Dimopoulos

**Affiliations:** W. Harry Feinstone Department of Molecular Microbiology and Immunology, Bloomberg School of Public Health, Johns Hopkins University, Baltimore, Maryland, United States of America; University of Melbourne, AUSTRALIA

## Abstract

*Anopheles gambiae* melanization-based refractoriness to the human malaria parasite *Plasmodium falciparum* has rarely been observed in either laboratory or natural conditions, in contrast to the rodent model malaria parasite *Plasmodium berghei* that can become completely melanized by a TEP1 complement-like system-dependent mechanism. Multiple studies have shown that the rodent parasite evades this defense by recruiting the C-type lectins CTL4 and CTLMA2, while permissiveness to the human malaria parasite was not affected by partial depletion of these factors by RNAi silencing. Using CRISPR/Cas9-based CTL4 knockout, we show that *A*. *gambiae* can mount melanization-based refractoriness to the human malaria parasite, which is independent of the TEP1 complement-like system and the major anti-*Plasmodium* immune pathway Imd. Our study indicates a hierarchical specificity in the control of *Plasmodium* melanization and proves CTL4 as an essential host factor for *P*. *falciparum* transmission and one of the most potent mosquito-encoded malaria transmission-blocking targets.

## Abstract 2

One way to block the spread of malaria is to modify the mosquito vectors so that they are unable to transmit the parasite. This study shows that the Anopheles mosquito can be engineered to block the human malaria parasite by melanizing it while in the mosquito’s midgut.

## Introduction

*Plasmodium falciparum* is the most prevalent malaria parasite in Africa, accounting for 99.7% of the 213 million malaria cases on that continent in 2018 [1]. A comprehensive understanding of the biology and transmission of this human-pathogenic parasite through its main mosquito vector, *Anopheles gambiae*, is paramount for developing new tools to control malaria. Anophelines are not passive vectors: They possess an effective innate immune system that controls infections with diverse microbes, including *Plasmodium* parasites, bacteria, and fungi, with some degree of specificity. The susceptibility of mosquitoes to *Plasmodium* and other pathogens, and, hence, vector competence, is an intricate process determined by a fine balance between antagonistic and agonistic immune mechanisms and factors [2]. Melanization, typically the deposit of a melanin layer on the pathogen surface that results in its encapsulation, is one of the most effective insect defense mechanisms, and extensive studies have shown that *A*. *gambiae* can melanize and thereby block infection with the rodent malaria parasite *Plasmodium berghei*.

Together with effector molecule-producing immune signaling pathways, the complement-like system acts as a key effector and regulator of *A*. *gambiae* immunity. The core of this system is an ensemble of hemolymph proteins, including the thioester-containing protein TEP1, which is activated by an unknown protease to form TEP1-cut, which is stabilized by the leucine-rich repeat immune proteins LRIM1 and APL1C, which coordinate its binding to the surface of microbes, leading to their elimination through lysis or melanization [3–9]. A complex cascade of serine proteases appears to regulate the TEP1-mediated pathogen killing. The CLIPA2 serine protease inhibits the TEP1-cut deposition on the microbial surface, while the CLIPA14 serine protease also protects *P*. *berghei* and other microbes from being melanized, acting more downstream in the process where it appears to regulate phenoloxidase activity [10,11]. Melanization reactions are also key players in *A*. *gambiae* immunity to bacteria and fungi and have also been shown to kill pathogens through lysis without the formation of a melanotic capsule [9,12–16]. The C-type lectins CTL4 and CTLMA2, which exist mainly as a heterodimer [13,17], have been described as host factors of the rodent parasite *P*. *berghei*, protecting the ookinete-stage parasites from the TEP1 complement-like system-regulated melanization, thus enabling them to develop into oocysts and ultimately into sporozoites that can infect the vertebrate host [7,18]. With regard to malaria parasites, most studies on the complement-like defense system have employed the rodent *P*. *berghei* model. Whether the same immune factors and mechanisms are involved in eliminating the clinically relevant human *P*. *falciparum* has not been clarified and is addressed in the present study.

We have previously used RNA interference (RNAi)-based gene silencing assays to show that partial depletion of *A*. gambiae CTL4 results in the melanization of a very small number of *P*. *falciparum* ookinetes, but only at unnaturally high infection intensities, and that CTL4 silencing does not affect overall mosquito susceptibility to the human malaria parasite, contrarily to the rodent *P*. *berghei*. Hence, previous studies by us and others did not prove a significant *P*. *falciparum* host factor role for *A*. *gambiae* CTL4, using human malaria laboratory strains and field isolates [19,20].

Here, we used CRISPR/Cas9 genome editing to knockout CTL4 and show that this factor is essential for the protection of the clinically relevant human *P*. *falciparum* parasite, through a mechanism that does not involve the known complement-like factors, which are important for *P*. *berghei* melanization [18–20]. Specifically, we show that the immune factors TEP1, LRIM1, and CLIPA2 do not influence melanization of *P*. *falciparum* NF54 ookinetes in CTL4^null^ mosquitoes while CLIPA14 does. The CTL4 partner, CTLMA2, plays a protective role for *P*. *falciparum* even when CTL4 is not present. Intriguingly, the human malaria parasite is not completely blocked by the CTL4-controlled melanization response while the rodent parasite is. Our study points at a significant influence of infection temperature, which is approximately 7 °C higher for *P*. *falciparum*, on the kinetics of midgut infection and efficiency of parasite melanization, enabling some human malaria parasites to escape this defense system. We also found that the key anti-*P*. *falciparum* innate immune pathway Imd does not influence parasite melanization, while it is known to mediate lysis-based anti-*P*. *falciparum* defense [21,22,23]. The CTL4^null^ mosquitoes are also highly refractory to the fungus *Beauvaria bassiana* through melanization, but more susceptible to bacterial infections, thereby pointing both agonistic and antagonistic roles of the C-type lectin complex for different pathogens [13,24]. We also show that the mosquito midgut microbiota marginally contributes to the CTL4^null^- refractoriness to *Plasmodium*. Our study proves a major role for CTL4 in the malaria mosquito biology and pathogen transmission and establishes CTL4 as a potent transmission-blocking target for the development of novel malaria control strategies.

## Results

### Generation and characterization of CTL4^null^ mutants

**Fig 1:**
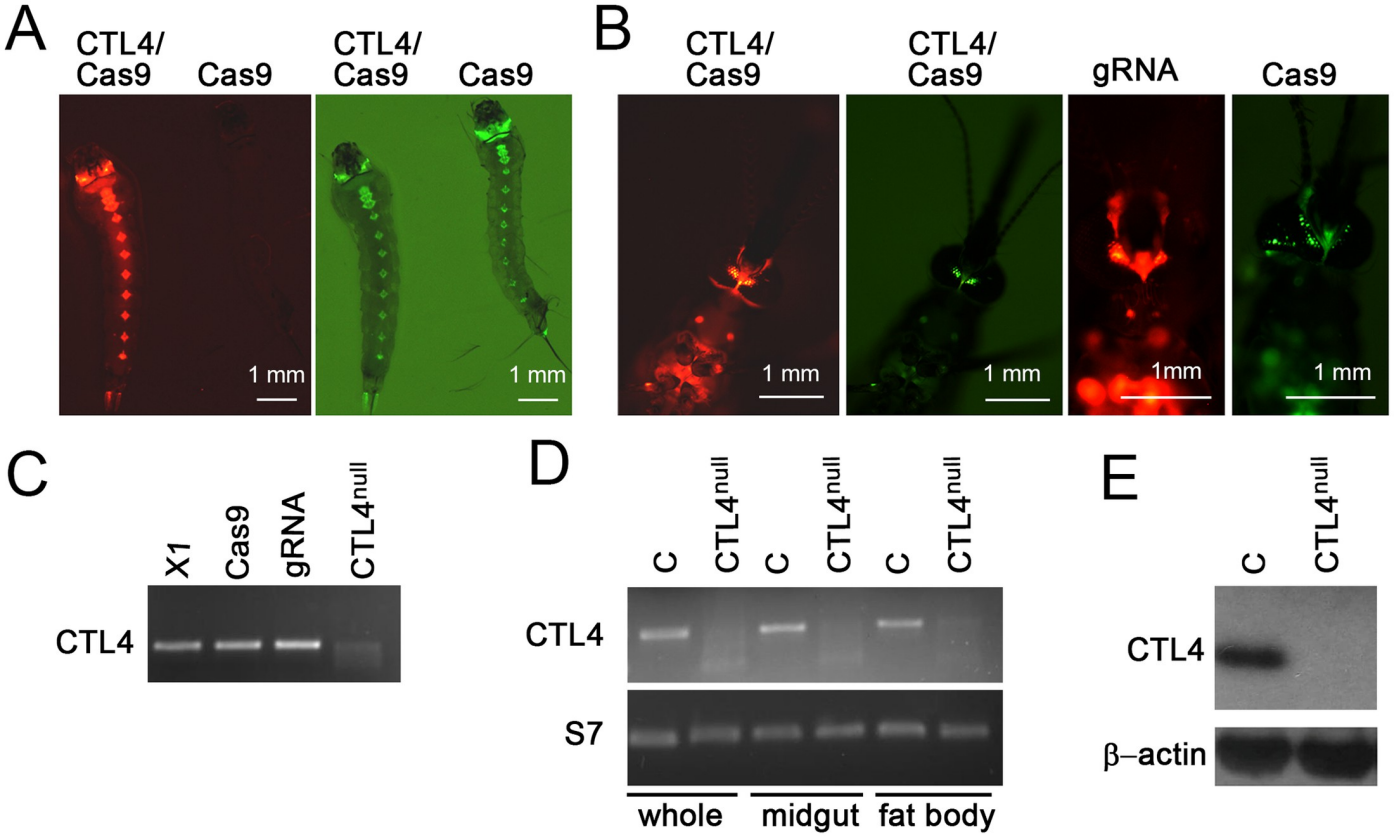
Generation of CRISPR/Cas9-mediated CTL4^null^ and confirmation of mutation. **(A, B)** CTL4-gRNA virgin females (RFP, red fluorescence) were crossed with Vasa-Cas9 males (YFP, yellow/green fluorescence) to produce *CTL4* gene knockouts (CTL4/Cas9) expressing both red and green fluorescence in the larval (**A**) and adult (**B**) stages. Progeny that did not inherit CTL4-gRNA (gRNA) expresses the Vasa-Cas9 green fluorescent marker only (Cas9). (**C)** PCR validation of *CTL4* gene deletion, compared to X1, Cas9, and gRNA controls (364 bp). Image of original gel is shown in S1 Raw Images. (**D)** Agarose gel image of the qRT-PCR products confirms CTL4 mRNA deletion (CTL4^null^) compared to the X1 control in different tissues (whole mosquito, midgut and fat body) (297 bp); expression levels were normalized to S7 (149 bp). Image of original gel is shown in S1 Raw Images. (**E)** Western blotting confirms CTL4 protein (15–20 kDa) deletion (CTL4^null^) as compared to the X1 in whole mosquitoes; expression levels were normalized to ß-actin (41 kDa). Images of original blots are shown in S1 Raw Images. gRNA, guide RNA; qRT-PCR, quantitative reverse transcription-PCR.

We have used our established CRISPR/Cas9 gene editing methodology [25] to generate *A*. *gambiae* CTL4-knockout mutant mosquitoes (CTL4^null^). We first created a transgenic *A*. *gambiae* line expressing 3 guide RNAs (gRNAs), each driven through a U6 snRNA polymerase III promoter, targeting the *CTL4* gene (S1 Fig). Individual gRNAs were first synthesized as short DNA linkers (S1 Table) and separately cloned into plasmid modules carrying the U6 promoter sequence followed by a gRNA expression template, and then assembled by a Golden Gate cloning reaction into the pDSAR transgenesis vector containing the 3xP3-RFP reporter [26] (S1 Fig). This construct with helper plasmid was microinjected into embryos of the *A*. *gambiae* X1 docking line, and transgenic progeny showing stable red fluorescence in the larvae were selected (S1 Fig). Following amplification of the *CTL4*-*gRNA*-expressing (CTL4-gRNA) transgenic population for at least 4 generations, a subpopulation enriched with homozygous transgenic mosquitoes was crossed with the *A*. *gambiae* Vasa-Cas9 strain to generate the CTL4-knockout (CTL4^null^) mutant mosquitoes (S1 Fig). Because of preadult stage fitness constraints when trying to generate and rear a homozygous population of knockout mosquitoes, we decided to use the CTL4-knockout mutant progeny of the crossing between CTL4-gRNA andVasa-Cas9 (gRNA/Cas9 transheterozygotes) for our studies, similarly to a previously published study [27]. Transheterozygotes from this cross carrying both transgenes were selected by their red and green fluorescent markers at the larval and adult stages (Fig 1A and 1B). The mutation/deletion in the adult mosquitoes was confirmed by both PCR with flanking primers (Fig 1C, S1 Table), quantitative reverse transcription PCR (qRT-PCR) in the various mosquito tissues (Fig 1D), as well as by sequencing of the PCR products (S1 File). The faint PCR products derived from the CTL4^null^ mosquitoes were also sequenced and shown not to correspond to CTL4 (S4 File). CTL4 protein deletion was also confirmed by western blot analysis, where no truncated CTL4 was observed (Fig 1E) using pools of 10 mosquitoes with a polyclonal serum raised against the mature CTL4. As a result of the *CTL4* mutation, the CTL4 protein was truncated from aa 121 (S1 File). These data show that CRISPR/Cas9-mediated disruption of CTL4 is complete in the gRNA/Cas9 transheterozygotes; therefore, the F_0_ somatic CTL4 knockout mutants were used as a model for further studies (referred to as CTL4^null^).

### CTL4 is a potent transmission-blocking target for malaria

**Fig 2:**
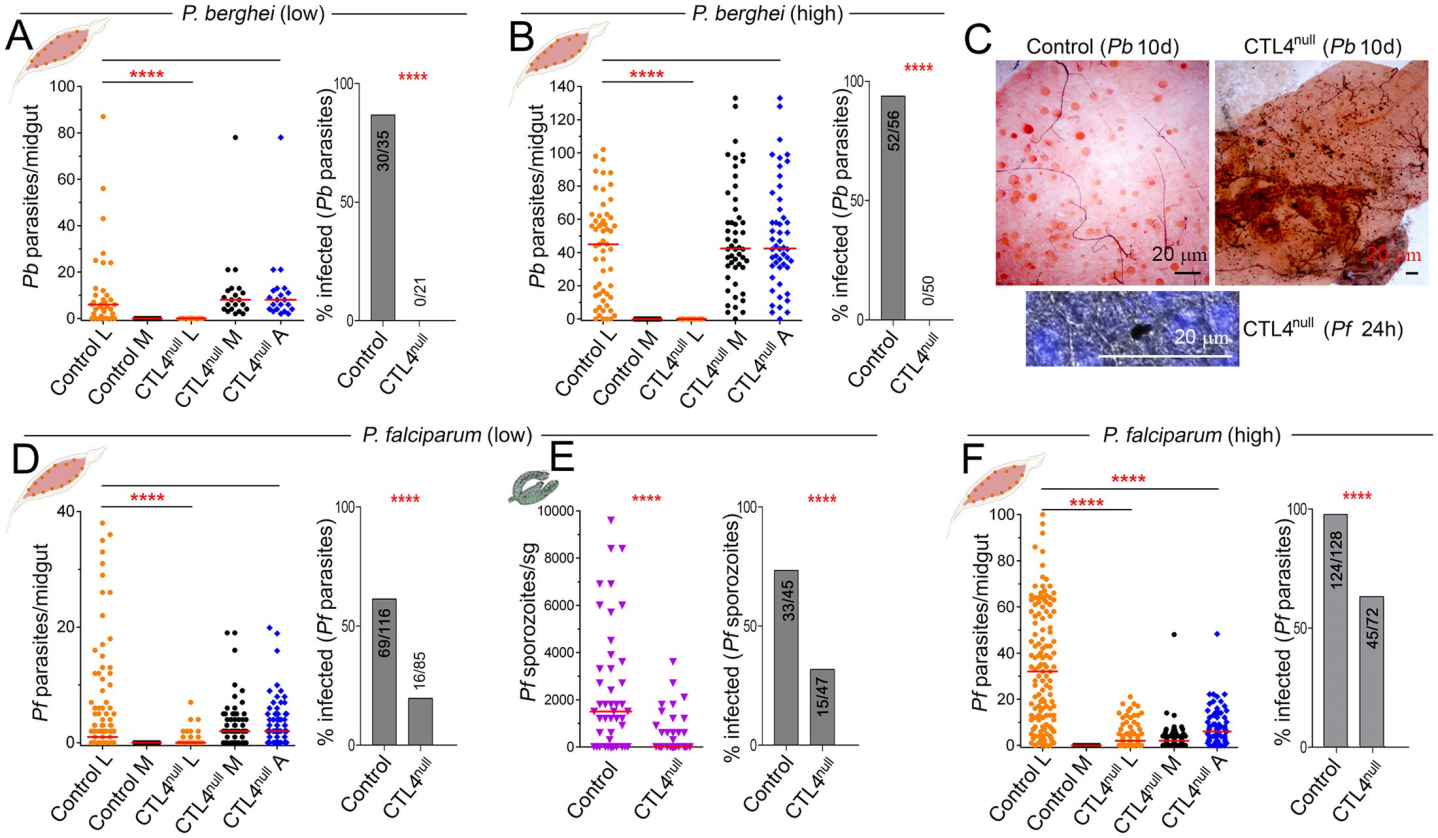
*Plasmodium* suppression in CTL4^null^ mosquitoes. **(A, B)** *P*. *berghei* infection intensity and prevalence in control and CTL4^null^ *A*. *gambiae* females fed on a mouse with low (**A**) or high (**B**) gametocytemia and measured at 10 dpi. (**C)** Images illustrate *P*. *berghei*-infected (high gametocytemia) *A*. *gambiae* control (upper left) and CTL4^null^ (upper right) midguts showing live parasites and 100% melanized parasites, respectively. Melanized *P*. *falciparum* ookinete (lower). (**D, E, F)** *P*. *falciparum* infection intensity and prevalence in control and CTL4^null^ *A*. *gambiae* females fed on blood with a low (**D, E**) or a high (**F)** gametocytemia and measured in the midgut at 8 dpi (**D, F**) or in the sg at 14 dpi (**E**). S5 File displays the underlying data. Dots and inverted triangles indicate the number of parasites in an individual midgut or salivary gland, respectively, and horizontal red bars indicate the median. Two-tailed *p*-values by Mann–Whitney test were used to compare the live parasites (L); M, melanized parasites (M), and all (L+M) parasites (A). Bars show the percentage of mosquitoes harboring at least one oocyst; the Fisher exact test was used to calculate *p*-values. Significance of parasite numbers: ****: *p* < 0.0001; horizontal black lines alone: not significant. dpi, days postinfection; sg, salivary glands.

First, we compared the susceptibility of all 3 control lines (CTL4-gRNA, the Vasa-Cas9, and the *A*. *gambiae* X1 docking line) to *P*. *berghei* and *P*. *falciparum* infections and found no significant differences among the 3 lines (S2 Fig), suggesting that either one of them can serve as a control. We therefore decided to use the X1 line as a control in subsequent experiments (“control” when used hereafter designates the X1 line). To assess the impact of CTL4 knockout on the susceptibility of *A*. *gambiae* to infection with the rodent *P*. *berghei* parasite, we fed CTL4^null^ and control (X1) mosquitoes on *P*. *berghei* (ANKA 2.34)-infected mice. As documented by several studies, *P*. *berghei* achieves unnaturally high infection intensities in *A*. *gambiae*, a consequence of *A*. *gambiae* not being its natural vector. Therefore, to overcome any possible parasite intensity-related dependence on the outcome of infection, we performed assays with *P*. *berghei* at both high and low infection intensities. Contrary to what we and others previously observed for RNAi-mediated silencing of *CTL4* in *A*. *gambiae* mosquitoes, the CRISPR/Cas9-induced knockout of this gene resulted in a complete (100%) refractoriness to both high and low *P*. *berghei* infection intensity (Fig 2A–2C) [7,15,18,20,28–30]. A strain-specific dependence to explain our results can be ruled out, since all the *A*. *gambiae* lines used in our study, including CTL4^null^, are derived from the G3 strain, which was also used in most of the studies on CTL4 mentioned above.

We next addressed the permissiveness of CTL4^null^ for the human malaria parasite *P*. *falciparum* (NF54). The CTL4-knockout mutants displayed strong *P*. *falciparum* suppression at low intensity infection, which mimicked the natural infections observed in the field (Fig 2D and 2E). The median oocyst count was significantly (*p* < 0.0001) reduced from 1 to 0 parasites/midgut, and the infection prevalence (mosquitoes harboring at least one parasite per total number of mosquitoes) was significantly (*p* < 0.0001) reduced from 61.3% to 19.7% in CTL4^null^ when compared to the control at 8 d post-blood meal (PBM) (Fig 2D and S2 Table). Interestingly, CRISPR/Cas9-mediated knockout of CTL4 resulted in the melanization of *P*. *falciparum* even at the low intensity infection, in contrast to studies based on RNAi-mediated CTL4 silencing, in which *P*. *falciparum* melanization was shown to be infection intensity-dependent and hence only observed with a high intensity infection [20]. Accordingly, CTL4 knockout also resulted in a profound decrease in sporozoite loads in the salivary glands of mosquitoes at 14 d PBM (Fig 2E and S2 Table). At a high intensity infection, CTL4-knockout mutants yielded a median of 2 oocysts/midgut, as compared to 32 for the control, and the prevalence of infection also significantly (*p* < 0.0001) decreased 2.2-fold, from 97.3% to 45.0% at 8 d PBM (Fig 2F and S2 Table). Conversely, at the high infection intensity, the total number of *P*. *falciparum* parasites (live and melanin-coated parasites combined) in the CTL4^null^ mosquitoes was significantly (*p* < 0.0001) lower than the number of total parasites in the control mosquitoes (Fig 2F). This discrepancy was not observed in the low *P*. *falciparum* infection intensity assay (Fig 2D) nor in *P*. *berghei* infection assays (Fig 2A and 2B), suggesting that CRISPR/Cas9-mediated disruption of CTL4 also results in the killing of *P*. *falciparum* without the formation of a melanotic capsule at high infection intensity.

In sum, we found that CTL4^null^ mosquitoes were completely refractory to *P*. *berghei* as a result of total melanization, and highly refractory to *P*. *falciparum* NF54, by melanization, or melanization in addition to another killing mechanism. This contrasts to the *A*. *gambiae* L3-5 genetically selected laboratory strain, which melanizes almost all rodent *P*. *berghei*, but is unable to melanize sympatric human malaria *P*. *falciparum* [31]. The development of *P*. *falciparum* was not completely blocked inCTL4^null^, indicating that the human malaria parasite is capable of partially evading the powerful defense system against which CTL4 protects it. While RNAi-based CTL4 gene silencing resulted in melanization of some parasites but not in a decrease of infection intensity and prevalence, the more extensive melanization and statistically significant reduction in viable human *P*. *falciparum* parasites in CTL4^null^corroborates the assertion that the gene silencing will most often yield a hypomorphic phenotype because of an incomplete depletion of target proteins [20]. The observation that the total number of *P*. *falciparum* parasites in the CTL4^null^ mosquitoes was significantly lower than the number in the control group suggests that CTL4 protects the human parasite not only from melanization but also from a killing mechanism that is either melanin formation independent or independent of the melanization process and manifests itself at higher levels of infection. Importantly, the level of *P*. *falciparum* suppression in CTL4^null^ mosquitoes was significantly higher than that achieved through deleting the *FREP1* host factor or by overexpressing the Imd pathway transcription factor *REL2* in *Anopheles*, indicating CTL4 as a powerful transmission-blocking target [25,21].

### CTL4 knockout-mediated *Plasmodium* killing occurs in the midgut epithelium

**Fig 3:**
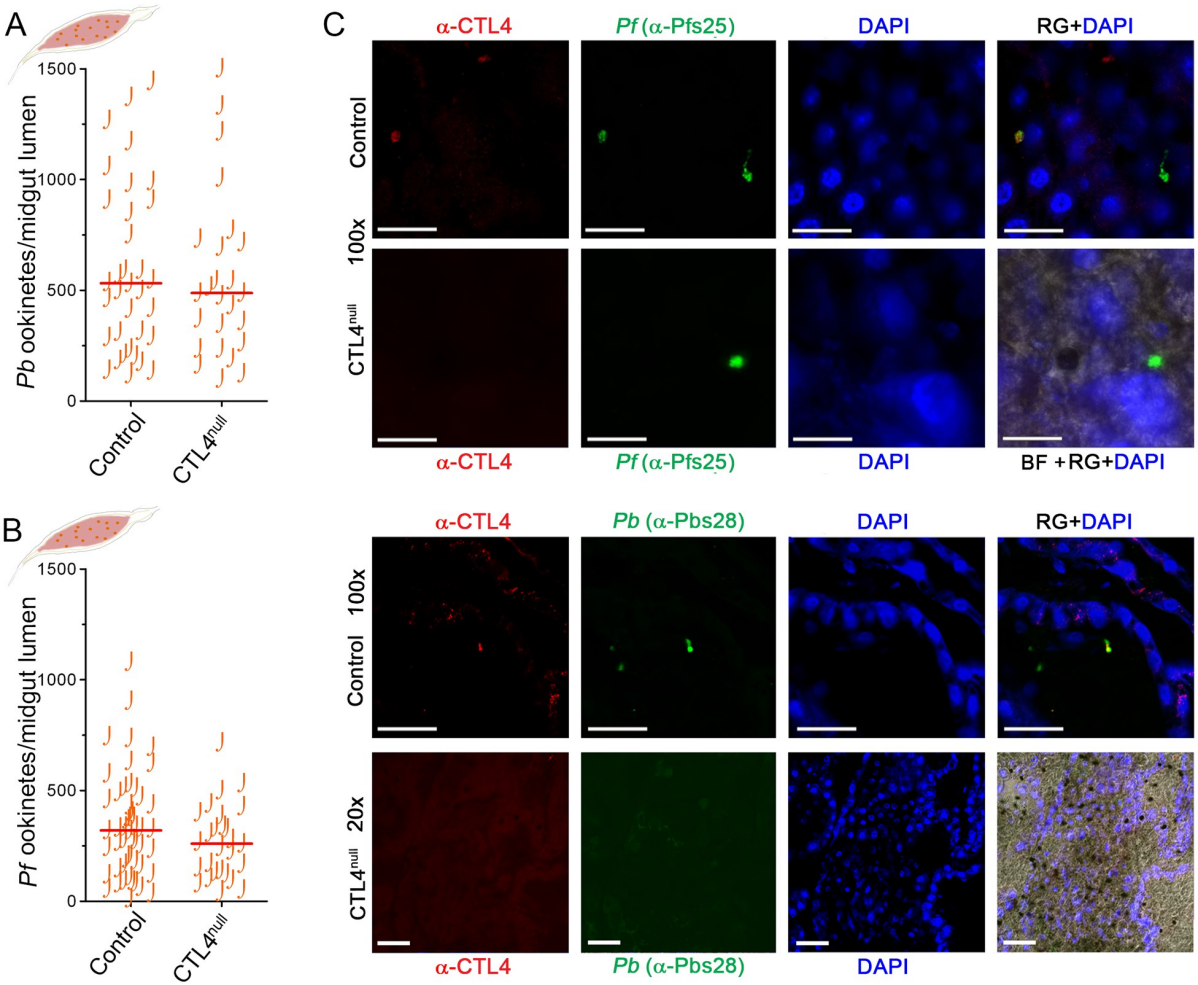
Spatial–temporal effects of CTL4 knockout on *Plasmodium* suppression in the *A*. *gambiae* midgut. **(A, B)** *P*. *berghei* (**A**) and *P*. *falciparum* (**B**) ookinete loads in the midgut lumen of *A*. *gambiae* females at 19 hpi were not significantly different between the control and the CTL4^null^ groups (two-tailed Mann–Whitney test). S5 File displays the underlying data. (**C)** Confocal microscopy images illustrate *P*. *falciparum*-infected (top panels) and *P*. *berghei*-infected (bottom panels) immunostained sections of the *A*. *gambiae* CTL4^null^ and control midgut epithelium at 24–25 hpi. Images are representative of 3 independent experiments with at least 10 midguts per replicate. Red indicates parasites probed with α-CTL4 antibody; green indicates live parasites probed with α-Pfs25 (for *Pf*) or α-Pbs28 (for *Pb*) antibodies; yellow indicates colocalization of CTL4 and the parasite; and blue indicates DAPI-stained epithelial cells nuclei. Black dots (CTL4^null^) represent melanized ookinetes. Scale bars 10 μm. hpi, hours postinfection.

Ookinete-stage *Plasmodium* invades the mosquito midgut epithelium beginning about 18 to 20 h after ingestion of infected blood, depending on parasite species. To address the question of whether the complete refractoriness of CTL4^null^ to *P*. *berghei* (Fig 2A–2C) occurs before or during midgut invasion, or at both stages, we measured the number of *P*. *berghei* ookinetes in the blood bolus at 19 h after ingestion, when the majority of parasites have not yet invaded the midgut epithelium. No differences were found in the lumen ookinete loads between CTL4^null^ and control mosquitoes (Fig 3A), indicating that the *P*. *berghei* decline in CTL4^null^ mosquitoes occurs when the parasite crosses the midgut epithelium. The peak of *P*. *berghei* ookinete invasion of *A*. *gambiae* occurs at 24 to 26 h postinfectious blood meal (hpi) [32]. Indeed, we found that all *P*. *berghei* ookinetes were melanized in the epithelium of CTL4^null^ mosquitoes at 25 hpi, while the epithelium of control mosquitoes displayed live anti-Pbs28-stained fluorescent parasites (Fig 3C), again showing that the CTL4-dependent *Plasmodium* protection takes effect exclusively during the invasion of the midgut epithelium.

We also wanted to investigate at which invasion stage the observed melanotic capsule-independent killing of *P*. *falciparum* occurs during high-intensity infection in CTL4^null^ mosquitoes (Fig 2F). As was true for the rodent parasite, the number of *P*. *falciparum* ookinetes in the lumen did not differ between the knockout and control cohorts (Fig 3B), indicating that CTL4 knockout-mediated *P*. *falciparum* NF54 lysis, together with melanization, takes place in the midgut epithelium. Melanized *P*. *falciparum* ookinetes were visible in the CTL4^null^ midgut epithelium at 24 to 25 hpi, together with live ookinetes (green) (Figs 2D–2F and 3C). Interestingly, while we observed colocalization of CTL4 with the parasite in control mosquitoes, the staining of CTL4 did not perfectly overlap with the contour of the parasite, suggesting that it might not be engaged in a direct interaction (Fig 3C).

### CTL4 knockout-mediated *Plasmodium* melanization is marginally promoted by the mosquito microbiota

**Fig 4:**
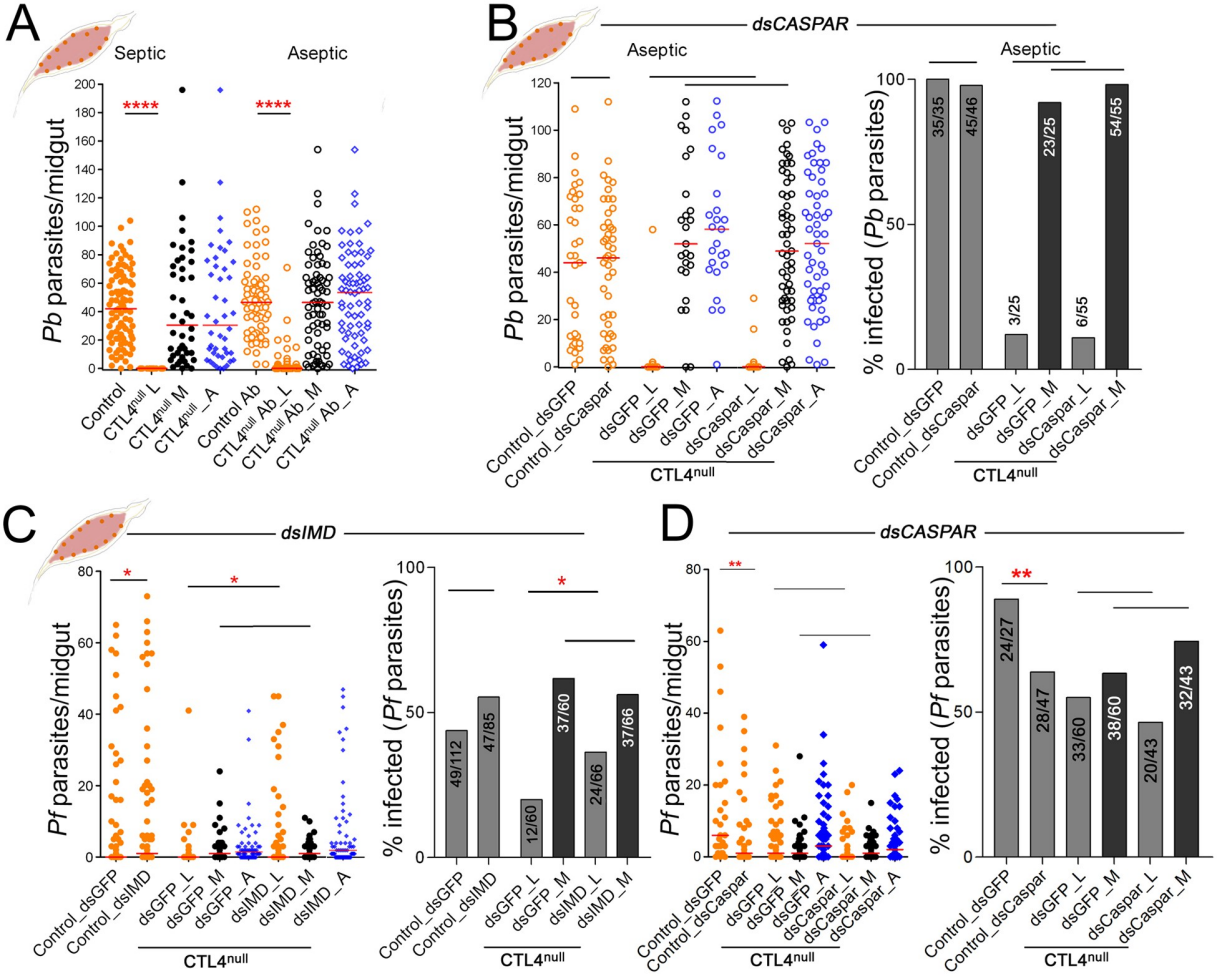
Effects of microbiota and Imd pathway on *Plasmodium* suppression in CTL4^null^ mosquitoes. **(A, B)** *P*. *berghei* infection in antibiotics-treated control and CTL4^null^ *A*. *gambiae* midguts at 10 dpi, compared to nonantibiotics treated (**A**) or after injection with dsCaspar or control dsGFP (**B**). (**C, D)** *P*. *falciparum* infection in control and CTL4^null^ *A*. *gambiae* females measured at 8 dpi, after RNAi-mediated silencing of *IMD* (**C**) and *Caspar* (**D**). S5 File displays the underlying data. Dots indicate the number of parasites in an individual midgut (L, live; M, melanized), and horizontal red bars indicate the median, compared by two-tailed *p*-values by Mann–Whitney test. Bars show the percentage of mosquitoes harboring at least one oocyst, and the Fisher exact test was used to calculate *p*-values. Significance of parasite numbers: *: *p* < 0.05, ****: *p* < 0.0001; horizontal black lines alone: not significant. dpi, days postinfection; RNAi, RNA interference.

Studies have shown that the *A*. *gambiae* melanization response can be triggered by bacteria in the mosquito hemolymph [12,13]. To exclude the possibility that the extreme phenotype of complete *P*. *berghei* melanization in CTL4^null^ mosquitoes is to some degree triggered by bacteria that can drive enzymatic cascades to reach the threshold needed to melanize ookinetes, we treated mosquitoes with an antibiotic cocktail to eliminate the majority of the bacteria from their midgut [22,33–35]. Interestingly, suppression of the bacteria in CTL4^null^ mosquitoes resulted in a few live *P*. *berghei* oocysts (median, 0 versus 46.5, respectively; *p* < 0.0001) (Fig 4A). These results demonstrate that the mosquito microbiota weakly promotes *Plasmodium* melanization in CTL4^null^ mosquitoes, possibly through the upregulation of immune factors or through bacteria-derived factors that can influence the process.

### CTL4-regulated *Plasmodium* melanization is independent of the Imd pathway

The immune deficiency (Imd) pathway is one of the major immune signaling pathways that is activated by, and controls, infections with both bacteria and *P*. *falciparum* in *A*. *gambiae*. We and others have shown that knockdown of its negative regulator, Caspar, confers a *P*. *falciparum*-resistant phenotype in *A*. *gambiae* based on parasite lysis but not melanization [22,23]. We have previously shown that the mosquito microbiota augments the Imd pathway [33], and our experiments with aseptic mosquitoes showed a lesser melanization of *P*. *berghei*; hence, we hypothesized that perhaps this was due to a modulation of the Imd pathway in the absence of immune-eliciting bacteria in the aseptic mosquitoes [21,33,35,36,37]. In order to investigate whether the Imd pathway could somehow be involved in the CTL4 knockout-induced *Plasmodium* melanization, we activated the Imd pathway by RNAi-mediated silencing of the pathway’s negative regulator *Caspar* (gene knockdown (kd) efficiency of 63.25%) in aseptic control and CTL4^null^ mosquitoes (Fig 4B) and compared the melanization phenotypes to mosquitoes treated with a control GFP double-stranded RNA (dsRNA). As shown in Fig 4B, Imd pathway augmentation in aseptic CTL4^null^ mosquitoes did not change the *P*. *berghei* melanization phenotype since some live oocysts would still form at the aseptic condition. Hence, the formation of some live *P*. *berghei* oocysts in CTL4^null^ mosquitoes at aseptic conditions is not due to a lack of a bacterially mediated modulation of the Imd pathway (Fig 4A and 4B). These data also suggest that the Imd pathway is not involved in CTL4 knockout-mediated melanization of *Plasmodium*. In *A*. *gambiae*, the Imd pathway has emerged as a key defense system against the human malaria parasite *P*. *falciparum* through a lytic killing mechanism (not melanization) [22,23]. To further explore a possible implication of the Imd pathway in the CTL4 knockout-induced melanization of *P*. *falciparum*, we independently silenced the *IMD* receptor protein gene (gene kd efficiency of 68.10%) and the negative regulator *Caspar* to inactivate and activate the pathway, respectively, prior to *P*. *falciparum* infection. Silencing *IMD* did not influence *P*. *falciparum* melanization in CTL4^null^ mosquitoes, whereas it did influence their susceptibility to parasite infection with live parasites in both the CTL4^null^ and control groups (Fig 4C), in agreement with our previous studies [22,23]. Silencing *Caspar* did also not influence the number of melanized *P*. *falciparum* ookinetes in the CTL4^null^ mosquitoes, corroborating the independence of the CTL4-regulated melanization from the Imd pathway (Fig 4D).

### CTL4-regulated *P*. *falciparum* melanization is temperature dependent

**Fig 5:**
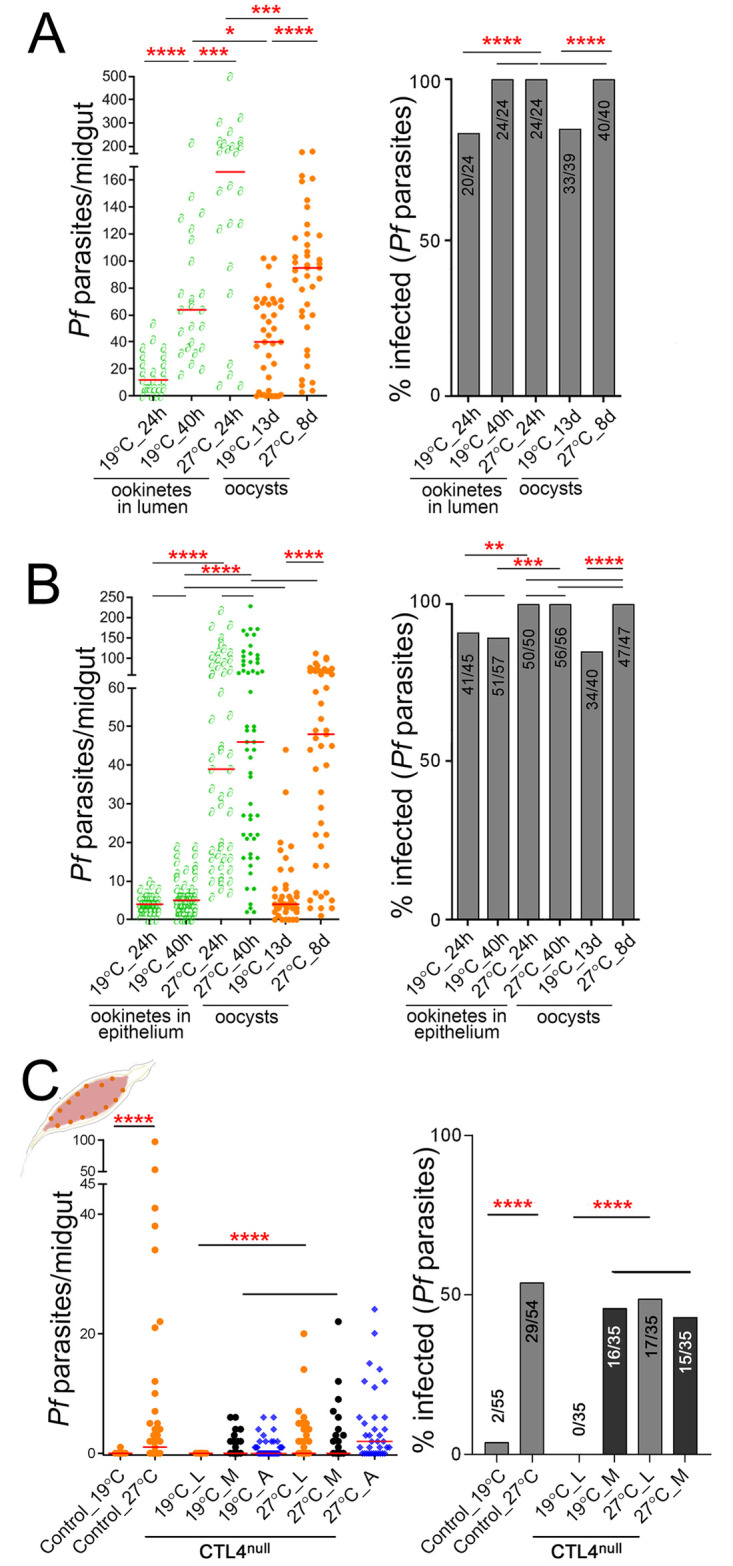
Influence of temperature on *Plasmodium* suppression in CTL4^null^ mosquitoes. **(A)** *P*. *falciparum* ookinetes in the lumen of control *A*. *gambiae* females at 24 and 40 hpi at 19 °C and 24 hpi at 27 °C, and oocysts at 13 dpi at 19 °C and 8 dpi at 27 °C. (**B)** *P*. *falciparum* ookinetes in the midgut epithelium of control *A*. *gambiae* females at 24 and 40 hpi at 19 °C and at 27 °C, and oocysts at 13 dpi at 19 °C and 8 dpi at 27 °C. (**C)** *P*. *falciparum* infection in control and CTL4^null^ *A*. *gambiae* females measured at 8 dpi at 19 °C or 27 °C. S5 File displays the underlying data. Dots indicate the number of parasites in an individual midgut (L, live; M, melanized), and horizontal red bars indicate the median, compared by two-tailed *p*-values by Mann–Whitney test. Bars show the percentage of mosquitoes harboring at least one oocyst, and the Fisher exact test was used to calculate *p*-values. Significance of parasite numbers: *: *p* < 0.05, **: *p* < 0.01; ***: *p* < 0.001; ****: *p* < 0.0001; horizontal black lines alone: not significant. dpi, days postinfection; hpi, hours postinfection.

As described above, *P*. *falciparum* infection in CTL4^null^ mosquitoes resulted in a powerful but somewhat leaky phenotype where the infection was not completely abrogated (Fig 1). Optimal infections of *A*. *gambiae* with *P*. *berghei* and *P*. *falciparum* occur at 19 to 20 °C and 27 °C, respectively, and this rather large temperature difference results in a slower rate of development/infection for the rodent parasite than for the human parasite within its vector [38,39]. We hypothesized that it might also influence immune response kinetics and exposure of the parasites to these immune responses, that could explain the complete versus incomplete melanization of *P*. *berghei* and *P*. *falciparum*, respectively, in CTL4^null^ mosquitoes. To determine whether the leaky *P*. *falciparum* infection phenotype of the CTL4^null^ mosquitoes could be attributed to the higher infection temperature, we performed *P*. *falciparum* infection experiments at both 19 °C and 27 °C with control and CTL4^null^ mosquitoes fed on the same *P*. *falciparum* NF54 gametocyte culture. First, we determined the effect of temperature on *P*. *falciparum* development and infection kinetics of the midgut lumen and epithelium in control mosquitoes. Reducing the temperature to 19 °C resulted in a dramatic reduction of ookinetes in both the midgut lumen and epithelium, as well as a significantly reduced number of formed oocysts (Fig 5A and 5B). In addition to the overall reduction of the various parasite stages at 19 °C, we also observed the expected slower midgut invasion and oocyst development kinetics (Fig 5A and 5B). Next, we assessed the effect of the lower temperature on *P*. *falciparum* melanization in CTL4^null^ mosquitoes. Interestingly, reducing the temperature to 19 °C did not affect the intensity and prevalence of parasite melanization compared to infection at 27 °C, but it completely abolished the formation of live oocysts in the CTL4^null^ mosquitoes (Fig 5C). These data show that melanization of *P*. *falciparum* at 19 °C is more efficient and extensive than that at 27 °C, since the proportion of melanized parasites in relation to the total number of ookinetes in the lumen and midgut epithelium, and live oocysts, is much larger at the lower temperature. These findings show that the observed differences in the proportions of melanized *P*. *berghei* and *P*. *falciparum* in CTL4^null^ mosquitoes is to a significant extent due to a difference in the temperature required for the sexual development of either parasite, as the more efficient melanization at the lower temperature is likely attributed to a slower invasion kinetics that prolongs the exposure of ookinetes to the mosquito’s melanization-mediated defense.

### CTL4 is a selective antagonist of bacterial infection

**Fig 6:**
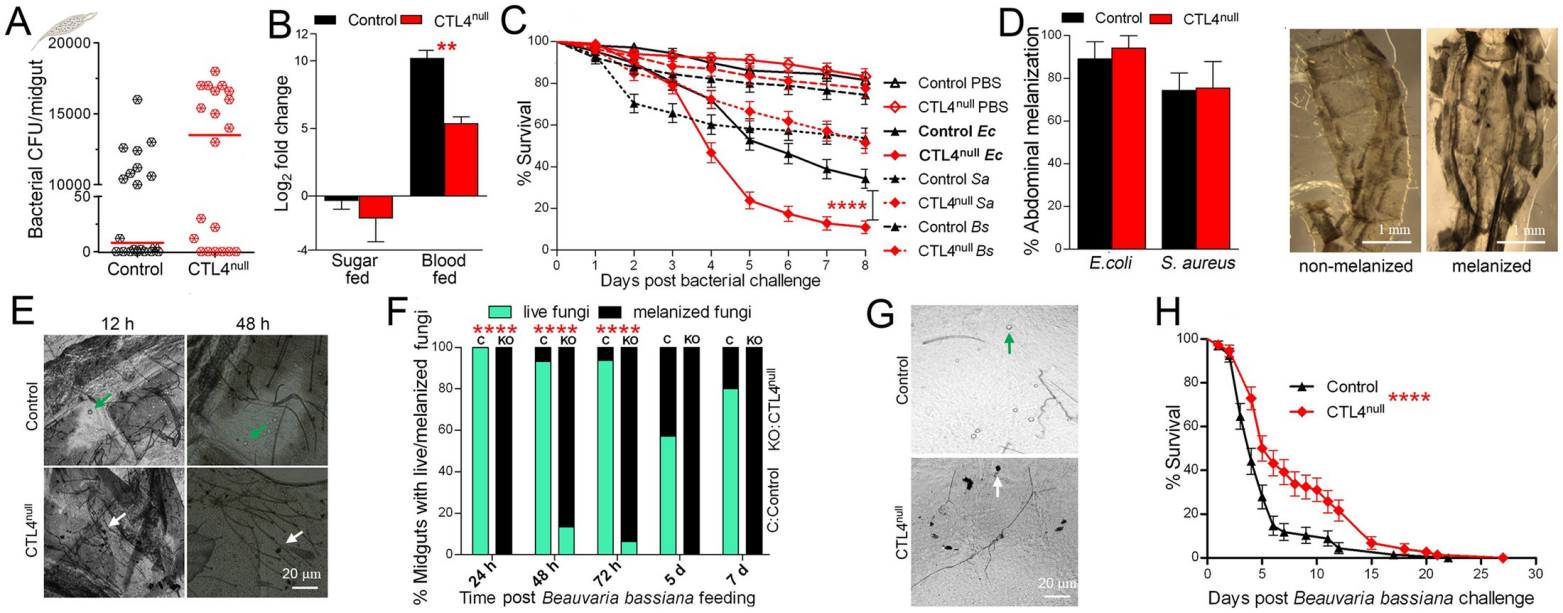
Influences of CTL4 on mosquito interactions with bacteria and fungi. **(A, B)** Midgut microbial flora of control and CTL4^null^ *A*. *gambiae* females was compared using two-tailed *p*-values by Mann–Whitney (**A**) and ANOVA followed by a Tukey test (**B**). (**C)** Control and CTL4^null^ *A*. *gambiae* females were injected with either *E*. *coli* (350,000 CFU), *S*. *aureus* (420,000 CFU), and *B*. *subtilis* (62,000 CFU), or PBS as a control, and longevity was analyzed up to 8 dpi. Kaplan–Meier survival analysis with a log-rank test was used to determine the *p*-values, and SEs of replicates are indicated. (**D)** Abdomens were dissected to check bacterial melanization 3 d following *E*. *coli* and *S*. *aureus* challenge by injection, and the Fisher exact test was used to calculate the difference between control and CTL4^null^ *A*. *gambiae* females. **(E)** Fungi spores were efficiently melanized (white arrows) in the CTL4^null^ abdomens 12 h and 48 h following injection of *B*. *bassiana* (2.15 × 10^6^ spores/ml), and the number of live spores (green arrows) was visibly higher in the control abdomens. (**F)** Melanized and live fungi were measured in midguts tissues from 24 h to 7 d after mosquitoes fed on a sucrose solution containing 2.15 × 10^8^ *B*. *bassiana* spores/ml, and the Fisher exact test was used to calculate the difference between control and CTL4^null^ *A*. *gambiae* females. (**G)** Melanized fungi were dectected on the midgut tissue of CTL^null^ females. (**H)** Survival of control and CTL4^null^ *A*. *gambiae* females after dipping (surface exposure) in *B*. *bassiana* spores (1 × 10^9^ spores/ml), compared by the Kaplan–Meier survival analysis with a log-rank test; SE of replicates are indicated. Significance: ****: *p* < 0.0001. S5 File displays the underlying data. CFU, colony-forming unit; dpi, days postinfection; SE, standard error.

Mosquito immune defense mechanisms have most likely principally evolved to combat infections with pathogens that are more prevalent and virulent than *Plasmodium* [40], such as bacteria and fungi, which are mainly present in the mosquito’s external environment and intestine. Hence, many of the immune genes and immune signaling pathways that mediate antibacterial and antifungal defenses are also involved in anti-*Plasmodium* immunity [2]. Knowing that CTL4 is essential for mosquito immunity/tolerance against bacterial systemic infections, we first investigated whether it also contributes to controlling the mosquito’s midgut microbiota. We first compared the number of Luria broth (LB)-culturable bacteria found in the midgut of either sugar-fed control and CTL4^null^ mosquitoes, and the results showed a marginally nonsignificant (*p* = 0.0612) increased bacterial load in CTL4-deficient mosquitoes (Fig 6A). To also capture contributions of nonculturable bacteria, we compared the total microbiota by qRT-PCR of bacterial 16s ribosomal RNA in both sugar-fed and blood-fed control and CTL4^null^ mosquitoes (Fig 6B). Results again showed that bacterial proliferation in the *A*. *gambiae* sugar-fed midgut is not significantly controlled by CTL4 but that CTL4 deletion affects the proliferation of bacteria following a blood meal, which was reduced in the CTL4^null^ mosquitoes, thereby suggesting that CTL4 has an agonistic effect on the midgut microbiota upon blood feeding through an unknown mechanism.

Next, we investigated the role of CTL4 in controlling systemic bacterial infections by monitoring the survival of CTL4^null^ and control mosquitoes after injection of either control PBS, gram-negative *Escherichia coli*, or gram-positive *Staphylococcus aureus* and *Bacillus subtilis* into the hemolymph. This assay showed a decreased survival of CTL4^null^ mosquitoes upon challenge with gram-negative *E*. *coli*, but not with the gram-positive bacteria (Fig 6C), in agreement with previous studies [13,24,32]. To investigate whether the increased CTL4^null^ mortality following *E*. *coli* challenge was related to bacteria-induced melanization, which could potentially produce toxic byproducts that are detrimental to the mosquito, we measured the mosquitoes’ abdominal melanization, which is visible in the cuticle 3 d after challenge with *E*. *coli* and *S*. *aureus* (Fig 6D). No differences were observed in the percentage of melanization between the CTL4^null^ and control cohorts, indicating that the decreased survival of *E*.*coli*-challenged CTL4^null^ mosquitoes was not due to a CTL4 knockout-induced increase in bacterial melanization (Fig 6D). Similarly, Schnitger and colleagues [13] did not observe an increase in phenol oxidase enzymatic activity in bacteria-challenged CTL4-silenced mosquitoes. In conclusion, our bacteria challenge assays suggest that CTL4 acts as an antagonist of systemic bacterial infections in a melanization-independent manner. It could be argued that the melanization observed in both CTL4^null^ and control mosquitoes in Fig 6C is a response to wounding provoked by the mechanical injection of bacteria, which equally affected both the CTL4^null^ and control cohorts. However, in contrast to another study in which thoracic melanization of the injection site was observed in CTL4-silenced mosquitoes but not in the ds*LacZ* control [15], we did not observe any increased abdominal melanization in our CTL4^null^ cohort(Fig 6D).

### CTL4 protects fungi from melanization

Mosquitoes are extensively exposed to fungi in their natural environments. To investigate whether CTL4 plays a role in mosquito immunity against fungi, we used the entomopathogenic fungus *Beauvaria bassiana*, which is virulent and has been shown to trigger the melanization response in *A*. *gambiae* [9,14,30]. After challenging mosquitoes with an intrathoracic injection of a *B*. *bassiana* spore solution, the number of live fungus spores was visibly higher in the abdomens of control mosquitoes as compared to the CTL4^null^ mosquitoes, in which the majority of the spores were efficiently melanized (Fig 6E). The CTL4^null^ mosquitoes also showed an increased melanization of *B*. *bassiana* associated with the midgut tissues, compared to the control cohort, at all assayed time points up to 7 d after feeding on a sterile sucrose solution containing *B*. *bassiana* spores (Fig 6F and 6G). Taken together, these assays demonstrate that CTL4 prevents melanization of fungi in both the *A*. *gambiae* abdomen and midgut tissues, suggesting that it plays a similar agonistic role for *B*. *bassiana* as it does for *Plasmodium*.

Although these experiments were helpful in elucidating the role of CTL4 in the *A*. *gambiae* immune response to fungi, the natural route of fungal infection in the mosquito does not require ingestion by the host (as for *Plasmodium* and bacteria) but rather consists of a direct penetration through the mosquito cuticle into the hemocoel after surface exposure. Therefore, we also monitored survival of CTL4^null^ and control mosquitoes after surface exposure by dipping mosquitoes in a *B*. *bassiana* spore solution. Interestingly, CTL4^null^ mosquitoes exhibited enhanced tolerance to *B*. *bassiana* infection, as reflected in their higher survival rate when compared to the control group (Fig 6H). The RNAi-mediated silencing of 2 key factors of the TEP1 complement-like system, *TEP1* and *CLIPA8*, has been shown to increase the susceptibility of mosquitoes to *B*. *bassiana* infection by reducing fungal melanization, revealing these 2 genes as positive regulators of fungal melanization [9]. Interestingly, both these immune proteins are also antagonists of *Plasmodium* infection, promoting parasite lysis and/or melanization [3,29]. The fact that CTL4 is an agonist/host factor and TEP1 and CLIPA8 are both antagonists/restriction factors of both *Plasmodium* and fungi indicates the existence of at least a partial overlap in the genetic modules that regulate the melanization response of *A*. *gambiae* to both microorganisms. It is therefore conceivable that CTL4 plays a broader role in protecting eukaryotic unicellular organisms. The enhanced tolerance of CTL4^null^ mosquitoes to *B*. *bassiana* infection is a particularly attractive feature that provides CRISPR/Cas9-mediated CTL4 knockout transgenic mosquitoes with a survival advantage over wild-type populations in the context of a fungal-based mosquito biocontrol strategy.

### CTL4 regulation of *Plasmodium falciparum* is independent of the TEP1 complement-like system

**Fig 7:**
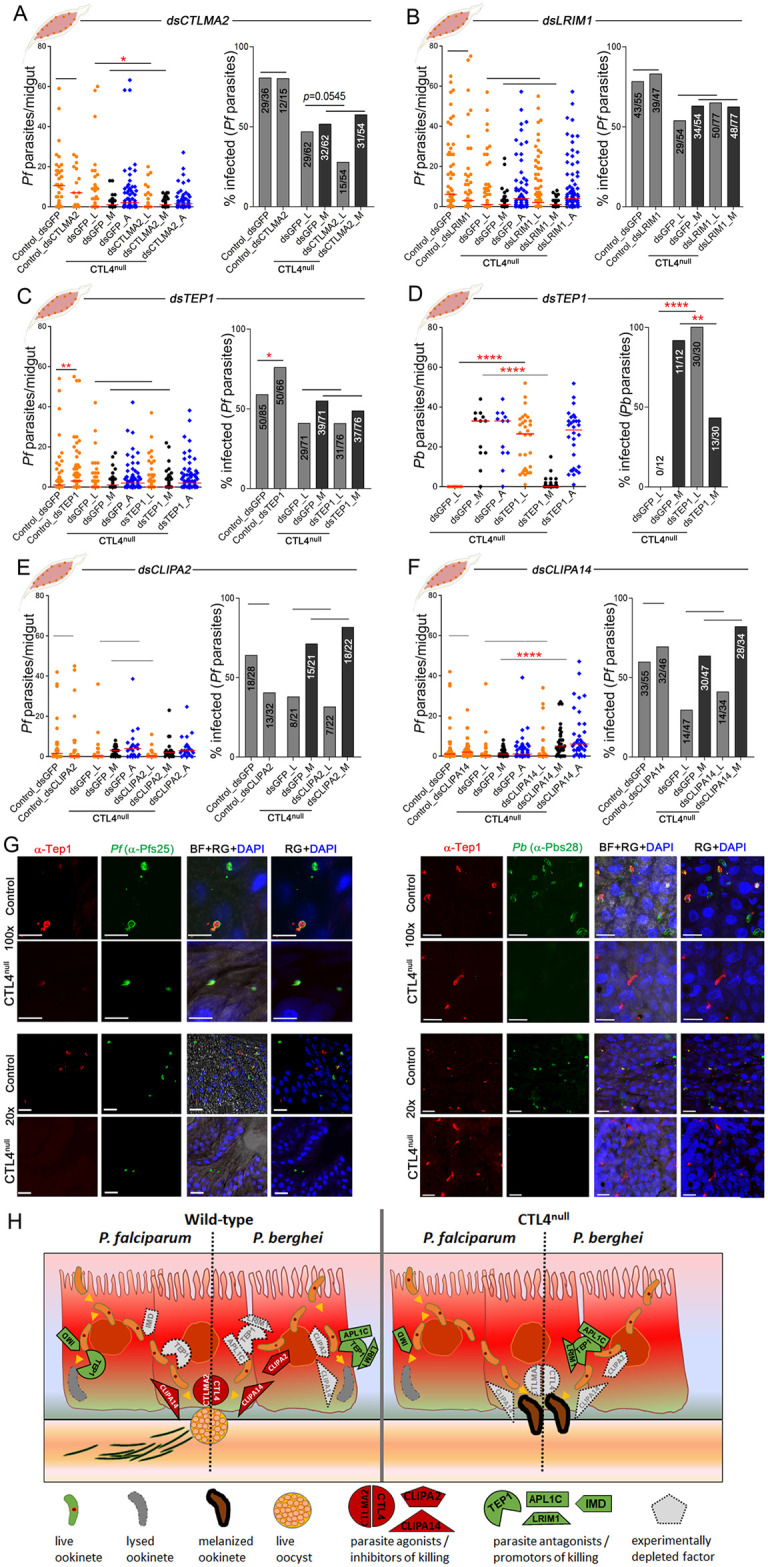
Interaction of *CTL4* with complement-like immune-related genes in *Plasmodium* suppression. **(A, B)** *P*. *falciparum* infection intensity and prevalence in control and CTL4^null^ *A*. *gambiae* females measured at 8 dpi, after RNAi-mediated silencing of *CTLMA2* (**A**) and *LRIM1* (**B**). (**C, D)** *P*. *falciparum* infection intensity and prevalence (**C**) measured at 8 dpi were compared to *P*. *berghei* (**D**) infection intensity and prevalence measured at 10 dpi, following RNAi-mediated silencing of *TEP1* in control and CTL4^null^ *A*. *gambiae* females. (**E, F)** *P*. *falciparum* infection intensity and prevalence in control and CTL4^null^ *A*. *gambiae* females measured at 8 dpi, after RNAi-mediated silencing of *CLIPA2* (**E**) and *CLIPA14* (**F**). S5 File displays the underlying data. Dots indicate the number of parasites (L, live; M, melanized) in an individual midgut, and horizontal red bars indicate the median, compared by two-tailed *p*-values by Mann–Whitney test. Bars show the percentage of mosquitoes harboring at least one oocyst, and the Fisher exact test was used to calculate *p*-values. Significance of parasite numbers: *: *p* < 0.05; **: *p* < 0.01; ****: *p* < 0.0001; horizontal black lines alone: not significant. (**G)** Confocal microscopy images illustrate *P*. *falciparum*-infected (left panels) and *P*. *berghei*-infected (right panels) immunostained sections of the *A*. *gambiae* CTL4^null^ and control midgut epithelium at 24–25 hpi. Images are representative of 3 independent experiments with at least 10 midguts per replicate. Red indicates parasites probed with α-TEP1 antibody; green indicates live parasites probed with α-Pfs25 (for *Pf*) or α-Pbs28 (for *Pb*) antibodies; yellow indicates colocalization of TEP1 and the parasite; and blue indicates DAPI-stained epithelial cells nuclei. Black dots (CTL4^null^) represent melanized ookinetes. Scale bars 10 μm. (**H)** Schematic representation of the influence of CTL4 and complement-like immune-related factors (those addressed/related to this study) on *P*. *berghei* and *P*. *falciparum*. From left to right: *P*. *falciparum* ookinetes are lysed by the action of the Imd pathway and TEP1 and are protected from being melanized by the CTL4/CTLMA2 complex and CLIPA14 in wild-type mosquitoes. When TEP1 and the Imd pathway are silenced, *P*. *falciparum* can develop into oocysts. *P*. *berghei* ookinetes are lysed by the action of TEP1, APL1C, and LRIM1, and they are protected from being lysed by CLIPA2 and CLIPA14 and are protected from being melanized by the CTL4/CTLMA2 complex, CLIPA2 and CLIPA14 in wild-type mosquitoes. When TEP1, APL1C, and LRIM1 are silenced, the *P*. *berghei* ookinetes can develop into oocysts. When CLIPA2 and CLIPA14 are silenced, the *P*. *berghei* ookinetes are lysed. In CTL4^null^ mosquitoes, the Imd pathway is promoting lysis of *P*. *falciparum* ookinetes, while silencing of CLIPA2 will enhance melanization of *P*. *falciparum* ookinetes. TEP1, APL1C, and LRIM1 are promoting melanization of *P*. *berghei* ookinetes in CTL4^null^ mosquitoes, and silencing of CLIPA2 and CLIPA14 will also promote melanization of *P*. *berghei* ookinetes in CTL4^null^ mosquitoes. dpi, days postinfection; hpi, hours postinfection; Imd, immune deficiency; RNAi, RNA interference.

We were interested in exploring the implication of other factors of the complement-like defense system in CTL4 knockout-dependent *Plasmodium* melanization. For this purpose, we used standard RNAi-based gene-silencing assays in conjunction with parasite infections. It is important to note that the RNAi-based approach used to silence genes achieves incomplete depletion of the target proteins and that a complete depletion would likely result in stronger phenotypes, similarly to what we have observed between our previous [20] and the present study. First, we addressed the C-type lectin CTLMA2, which forms a dimer with CTL4 and yields a similar phenotype when silenced by RNAi in wild-type mosquitoes [13,18–20]. While no effect on *P*. *falciparum* infection was observed between the *CTLMA2*-silenced (gene kd efficiency of 87.02%) and the GFP dsRNA-treated control mosquitoes (Fig 7A), silencing this gene in CTL4^null^ mosquitoes resulted in a marginal reduction of live oocysts when compared to GFP-treated mosquitoes, but no change in ookinete melanization, suggesting that CTLMA2 also protects against melanotic capsule-independent parasite killing (Fig 7A). Previous studies have suggested that CTL4 and CTLMA2 are secreted into the hemolymph in the form of a disulfide-linked heterodimer [13,17], thus explaining their similar roles in the mosquito defense against bacteria and *Plasmodium*. However, our results (Fig 7A) reveal a minor agonistic role for CTLMA2 that is independent of CTL4. These results suggest that in the context of the *A*. *gambiae* immune response to the human malaria parasite, CTLMA2 may still be moderately active as a monomer or homodimer [13] that functions differently from the CTL4-CTLMA2 heterodimer.

The LRIM1/APL1C complex is a *Plasmodium* antagonist required for the binding of TEP1 to ookinetes [4–7] and hence acts upstream of CTL4. Several studies using RNAi gene silencing have demonstrated that the leucine-rich repeat protein LRIM1 is necessary for *P*. *berghei* melanization in *A*. *gambiae* [6,18]. To investigate the implication of LRIM1 in *P*. *falciparum* melanization, we silenced LRIM1 (gene kd efficiency of 80.96%) and observed no variation in the number of live parasites in either the control or the CTL4^null^ cohorts, or melanized parasites in the CTL4^null^ mosquitoes (Fig 7B), thereby showing that LRIM1 is not necessary for the CTL4 knockout-mediated melanization of *P*. *falciparum* NF54.

TEP1 is one of the most studied factors in the *A*. *gambiae* anti-*Plasmodium* defense as a *Plasmodium* antagonist that provides protection against both human and rodent malaria parasites, triggering immune responses that include lysis and melanization [3,7,22,32,41]. TEP1 is acting upstream of a cascade of clip-domain serine proteases and leucine-rich domain proteins that control lysis and melanization of *P*. *berghei* and *P*. *falciparum*, and melanization of *P*. *berghei* in absence of CTL4 [7]. It has been proposed that some *P*. *falciparum* strains have partly adapted to geographically sympatric vectors through evasion of complement-like immune responses [41]. Nevertheless, the *P*. *falciparum* NF54 strain does not evade TEP1-mediated lysis completely, as shown by us and others in *TEP1*-silencing experiments [32,22,42]. Interestingly, TEP1 knockdown (gene kd efficiency of 79.32%) did not abolish melanization-based resistance to *P*. *falciparum* in CTL4^null^ mosquitoes, but it did reverse the lysis-mediated killing of this parasite in the control mosquitoes (Fig 7C). This result is in sharp contrast to the *P*. *berghei* phenotype seen in CTL4^null^ mosquitoes, where RNAi-mediated silencing of TEP1 abolishes total refractoriness and decreases the number of melanized *P*. *berghe*i parasites (Fig 7D), in a manner reminiscent of earlier findings [7]. Hence, the CTL4^null^ *P*. *falciparum* resistance phenotype is both TEP1 and LRIM1 independent. Consistent with this observation, confocal microscopy revealed that TEP1 (red) colocalizes with dead *P*. *berghei* ookinetes at 24 to 25 hpi in CTL4^null^ mosquito midguts (Fig 7G, right panels). However, its colocalization with *P*. *falciparum* ookinetes is faint or nonexistent in CTL4^null^ mosquitoes, in contrast to control mosquitoes (Fig 7G, left panels, S3 Table). In fact, only 43% of the *P*. *falciparum*-infected CTL4^null^ midguts from 3 biological independent replicates showed TEP1-positive ookinetes, with weak TEP1 staining (Fig 7G, left panels, S3 Table), as compared to 75% of the control midguts, thus confirming the lack of TEP1–*P*. *falciparum* interaction when CTL4 is knocked out.

A CLIP domain serine protease cascade regulates TEP1-dependent *P*. *berghei* melanization, and both CLIPA2 and CLIPA14 are negative regulators (antagonists) of this process [10,11]. Silencing CLIPA2 (gene kd efficiency of 87.45%) in CTL4^null^ mosquitoes did not alter the *P*. *falciparum* infection phenotype (Fig 7E), while silencing CLIPA14 (gene kd efficiency of 91.04%) increased the number of melanized *P*. *falciparum* NF54 ookinetes (Fig 7F), yielding a phenotype similar to that with *P*. *berghei* infection [11].

## Discussion

Here, we reveal the role of CTL4 in immunity against the human malaria parasite and against fungi and bacteria using CRISPR/Cas9-generated CTL4^null^ *A*. *gambiae* mosquitoes. We show that CTL4’s protective role is exclusively exerted in the mosquito midgut epithelium on invading ookinetes, and, unlike the murine parasite *P*. *berghei*, the human malaria parasite *P*. *falciparum* is not completely melanized in CTL4^null^ mosquitoes. This partial refractoriness is to a significant extent related to the higher temperature of *P*. *falciparum* infections, which allows this parasite to develop and traverse the midgut epithelium quicker than the rodent parasite at the lower temperature (Fig 5). It is possible that other differences in the biology of *P*. *berghei* and *P*. *falciparum* infections in *A*. *gambiae* also relate to the 7 °C difference in infection temperature. While the complete removal of CTL4 in CTL4^null^ mosquitoes resulted in strong melanization phenotypes and significant suppression of both *P*. *berghei* and *P*. *falciparum*, its partial depletion through RNAi-based silencing only yielded strong melanization and suppression of *P*. *berghei* in *A*. *gambiae* [20]. This suggests that the rodent parasite has a higher dependence on the quantity of available CTL4, most likely because of the slower rate of invasion of the midgut epithelium, while the faster migrating *P*. *falciparum* may not enable the melanization-mediated defense system to target it as effectively.

Importantly, our study demonstrates that the functions of key anti-*Plasmodium* effectors in the defense against the human malaria parasite differ from those acting against the rodent counterpart. The TEP1 and LRIM1 effectors, and the CLIPA2 agonist, which regulate the melanization of *P*. *berghei*, do not influence *P*. *falciparum* NF54 melanization. TEP1 does also not bind to the surface of the *P*. *falciparum* ookinete, in contrast to the mechanism regulating *P*. *berghei* melanization (Fig 7G). However, the serine protease CLIPA14, which is further downstream in the molecular cascade leading to melanization, influences *P*. *falciparum* NF54 melanization similarly to *P*. *berghei* melanization. Taking our findings together with previous studies, it becomes apparent that the parasite species specificity of this molecular cascade is higher upstream, where *A*. *gambiae* TEP1, LRIM1, and CLIPA2 are specific for the regulation of *P*. *berghei* killing and melanization, while the species specificity decreases downstream, as CLIPA14, CTL4, and CTLMA2 regulate melanization of both parasite species (Fig 7H) [10,11,20]. It is quite likely that other unknown members of the TEP, LRIM, and CLIP families may be implicated in regulating *P*. *falciparum* melanization.

We also show that CTL4 knockout-mediated killing of *P*. *falciparum* is not exclusively the result of capsule-dependent melanization, since more than 50% of the ookinetes appeared to be killed without the formation of a melanotic coating of the parasites in CTL4^null^ mosquitoes at a high intensity of parasite infection. This killing mechanism is likely the result of an incomplete melanization reaction that does not result in the formation of a melanotic capsule, as shown for *Drosophila* [16]. Interestingly, silencing of *CTLMA2*, which resulted in a decreased number of live *P*. *falciparum* oocysts in the CTL4^null^ mosquitoes, was not accompanied by an increase in ookinete melanization, suggesting that CTLMA2 may protect the parasite from additional nonmelanization-related killing mechanisms and is not exclusively exerting its function as an interacting partner with CTL4. While the Imd pathway is a key anti-*P*. *falciparum* lysis-based defense system, it does not influence melanization of *Plasmodium* in CTL4^null^ mosquitoes. It will be interesting to address the effects of CTL4 knockout on different malaria parasites strains in future studies.

Mosquitoes are continuously exposed to a variety of bacteria and fungi, and our infection assays with CTL4^null^ mosquitoes show that CTL4 is also an agonist of fungal infections through a melanization-based defense similarly to that mounted against *Plasmodium*, but an antagonist of systemic bacterial infections. The opposite roles of CTL4 in fungal and bacterial systemic infections are particularly interesting; like fungi, *Plasmodium* is also unicellular eukaryote, in contrast to bacteria being prokaryotes with a cell wall. Our results suggest that both *Plasmodium* and *B*. *bassiana* recruit CTL4 as a protective factor against melanization-based killing. The mosquito’s natural midgut microbiota is weakly promoting *Plasmodium* melanization, likely though priming of additional immune factors, but not the antibacterial and anti-*Plasmodium* Imd pathway.

The CTL4 agonist system described in this study is particularly interesting as a malaria transmission-blocking target because it achieves a level of refractoriness to the human malaria parasite that we have not observed with other genetically modified *Anopheles* lines, without compromising the life span of adult mosquitoes (S3 Fig). Modeling studies have shown that total transmission blocking, or refractoriness, is not necessary to achieve an epidemiologically significant impact on disease prevalence and that even a 35% transmission-blocking effect would result in malaria elimination from a hypoendemic area [43,44]. Furthermore, studies using *Anopheles stephensi* and *Plasmodium yoelii* infection models, whose sporozoite infectivity is similar to that observed for *P*. *falciparum*, have shown that the likelihood of transmission is significantly decreased at low sporozoite loads (<10,000 sporozoites) in the salivary glands [45]. Our CTL4^null^ mosquitoes exceeded a 35% reduction in *P*. *falciparum* infection prevalence, and the salivary gland sporozoite loads were strongly suppressed, predicting a significant impact on malaria transmission as a result of a population replacement of wild-type with CTL4-deficient mosquitoes. However, the adverse effects of a CTL4 germline-based knockout on embryonic development call for alternative approaches to inactivate CTL4 function at the life stage and in the tissues that are relevant for *Plasmodium* development. *B*. *bassiana* is also an entomopathogenic fungus that has been developed into an ecologically friendly biopesticide for mosquito control [14,46–50]. The increased resistance of CTL4-deficient mosquitoes to both *Plasmodium* and *B*. *bassiana* could thereby be exploited to provide a competitive/selective advantage to the malaria-refractory CTL4^null^ mosquitoes against their wild-type counterparts through *B*. *bassiana* exposure.

## Materials and methods

### Ethics statement

All animal work was conducted in strict accordance with the recommendations in the Guide for the Care and Use of Laboratory Animals of the National Institutes of Health (NIH), USA. The protocols and procedures used in this study were approved by the Animal Care and Use Committee of the Johns Hopkins University (Permit Number: MO18H82) and the Johns Hopkins School of Public Health Ethics Committee. Commercial anonymous human blood was used for parasite cultures and mosquito feeding; thus, informed consent was not required.

### Generation of U6-gRNA constructs

Putative gRNA sequences targeting the *A*. *gambiae* CTL4 (AGAP005335) gene were identified using ZiFiT (zifit.partners.org). Sequences with no off-target sites, 20 nucleotides long, starting with a guanine (G) to facilitate transcription by the U6 promoter, and followed by a protospacer adjacent motif (PAM; NGG, where N is any nucleotide), were chosen (S1 Table). The final selected gRNA target sequences are within the region encompassing the CTL4 RNAi primers. For each of the 3 gRNA targets selected for the CTL4 gene, we designed a forward and a reverse primer, complementary to each other (S1 Table). To generate each of the three pKSB-gRNA vectors (pKSB-CTL4-gRNA-1, -2, -3), each of the 3 double-stranded DNA fragments was individually cloned into a pBluescript vector backbone that contained the U6 snRNA polymerase III promoter (AGAP013557), CRISPR RNA invariable sequences, and the RNA polIII TTTTT terminator (as in [25]), followed by sequencing confirmation. The pKSB-CTL4-gRNA vectors were used to assemble 3 gRNA copies of CTL4 into a destination pDSAR vector via the Golden Gate assembly system (New England Biolabs, E1600S) for embryo microinjection of the *A*. *gambiae* docking line X1 (S1 Fig) [26]. The final plasmid, pDSAR-CTL4-gRNA3 (with 3xP3-RFP), allowed screening of gRNA-positive larvae or adult mosquitoes by using the red fluorescent eye marker (Fig 1, S1 Fig).

### Mosquito rearing and embryo microinjection

All the *A*. *gambiae* s.s. mosquito lines used in this study were derived from the G3 strain, including the docking line X1 as well as the Vasa2-Cas9 [51] and CTL4-gRNA3 lines described above, here referred to as Cas9 and CTL4-gRNA, respectively. Mosquitoes were reared and maintained under laboratory conditions according to Simões and colleagues [52]. Embryo microinjection solution was prepared by combining maxi-prep purifications (Invitrogen) of 2 plasmids: the pDSAR-CTL4-gRNA3 construct (160 ng/μl) and a helper plasmid, pENTR-R4R3-Vasa2-integrase (200 ng/μl), expressing the phiC31 integrase under the Vasa promoter [26,53], in 1/10 of 10× injection buffer solution (0.1 mM NaHPO4 buffer and 5 mM KCl (pH 6.8)).

Approximately 700 *A*. *gambiae* X1 docking line embryos were injected [26,54] and maintained on wet filter paper for 2 d before hatching [25]. The hatched G_0_ larvae were screened for transient red fluorescence expression at the second instar larval stage under a fluorescence stereomicroscope. Approximately 29.5% of the hatched larvae showed RFP fluorescence, and the RFP+ G_0_ were sexed at pupal stage and emerged adults were crossed with X1 adults from the opposite sex (virgin females and males were prepared in the same way as the gRNA line). The G1 progeny were examined for RFP fluorescence at both the larval and adult stages. Positive G1 mosquitoes were outcrossed with X1 for 2 generations, followed by 2 generations of self-crossing to enrich the homozygous CTL4-gRNA mosquitoes by the screening of RFP in the larvae.

### Generation of CRISPR/Cas9 CTL4-knockout mutants

Nearly fully homozygous CTL4-gRNA mosquitoes starting from generation 5 were crossed with the Vasa-Cas9 strain to generate CTL4-knockout (CTL4^null^) mutants (S1 Fig). Next, we sought to obtain a line of homozygous CTL4^null^ mosquitoes by crossing the CTL4 knockout mutants with X1 mosquitoes, but the progeny of these crossings resulted in either nonhatching eggs or only wild-type descendants, as described in Yang and colleagues [27]. Given this challenge, we decided to use the CTL4^null^ mosquitoes, which are the transheterozygous progeny of the crossing of the CTL4-gRNA virgin females with the Vasa-Cas9 males (S1 Fig), for all our assays. All generations of CTL4^null^ mosquitoes were screened for both red and green fluorescence (Fig 1). The mutation/deletion in CTL4^null^ females was assessed by PCR with flanking primers and confirmed by qRT-PCR in the different mosquito tissues (Fig 1). The faint PCR products derived from CTL4 ^null^ were also sequenced by Sanger sequencing, after gel excision and purification, showing that they do not contain CTL4 sequences (S4 File). The mutation of the *CTL4* gene was determined by alignment of the mutant sequence with the wild-type sequence (S1 File). All primers sequences used are listed in S1 Table.

### CTL4 and Tep1 antibodies and western blotting

Polyclonal CTL4 and TEP1 antibodies were generated by Boster Biological Technology in rabbit targeting antigens corresponding to peptide sequences of the 2 genes (cDNA sequences used are available in S2 and S3 Files). Control and CTL4^null^ 3 pools of 10 mosquitoes were homogenized in PBS with protease inhibitor cocktail (cOmplete, EDTA-free Protease Inhibitor Cocktail, Roche), and samples were stored overnight at 4 °C. Protein concentrations were measured with a Micro BCA Protein Assay Kit (Thermo Fisher Scientific). Western analysis was done following a previously established protocol with modifications [55]. About 20 μg of total protein were mixed with NuPAGE LDS Sample Buffer (4X) (Thermo Fisher Scientific) and incubated at 95 °C for 10 min before they were separated on a 4% to 20% SDS-PAGE gradient gel (Novex WedgeWell 4% to 20%, Tris-glycine, 1.0-mm Mini Protein Gel, 10-well, Thermo Fisher Scientific). Proteins were transferred to a nitrocellulose membrane through a Trans-Blot Turbo Transfer System (BioRad), following incubation in blocking buffer (1X PBS with 0.05% Tween-20, 5% nonfat milk powder) overnight at 4 °C. Anti-*A*. *gambiae* CTL4 rabbit polyclonal antibody was diluted 1:500 in blocking solution and incubated for 1 h. The blot was washed 3 times for 15 min each with 1xPBS (Tween-200.05%), incubated with HRP-linked anti-rabbit IgG secondary antibody (Cell Signaling Technology, 7074) at 1:15,000 dilution for 1 h, washed again, and developed using ECL Prime Western Blotting Detection Reagent (GE Healthcare). In addition to sample normalization with the BCA protein assay, the membrane was stripped for 20 min with Restore Plus Western Blot Stripping Buffer (Thermo Scientific), blocked and incubated with anti-beta actin primary antibody-loading control (Abcam, 8224) at 1:1,000 dilution, followed by HRP-linked anti-mouse IgG secondary antibody (Cell Signaling Technology, 7076) at 1:15,000 dilution.

### *Plasmodium* infection and longevity assessment

To assess anti-*Plasmodium* activity, female mosquitoes were fed on an anesthetized *P*. *berghei* (ANKA 2.34 strain)-infected mouse or through artificial membrane feeders on an NF54 *P*. *falciparum* gametocyte culture in human blood. For high-infection *P*. *berghei* experiments, female mosquitoes were allowed to feed for a longer period on mice with higher parasitemia. High (approximately 0.1%) or low (approximately 0.01%) gametocytemia was used for *P*. *falciparum* NF54 infection experiments. Unfed females were removed, *P*. *berghei*- and *P*. *falciparum*-infected mosquitoes were kept at 19 °C or at 27 °C, respectively, except for some *P*. *falciparum*-infected cohorts that were kept at 19 °C for temperature-dependent experiments. At 19 h postinfection (hpi), infected midguts were dissected in PBS, the blood bolus was removed, and the midguts were homogenized and transferred to a glass slide on which they were fixed in methanol. Midguts were then stained with Giemsa, and ookinetes in the midgut lumen were counted using a Leica DM 2500 microscope. For temperature-dependent experiments with the control mosquitoes, the lumen ookinetes were counted using the blood bolus at both 24 hpi and 40 hpi for 19 °C and 24 hpi for 27 °C treatments through Giemsa staining. The epithelium ookinetes at 24 hpi and 40 hpi for 19 °C treatment, and 24 hpi ookinetes and 40 hpi early oocysts for 27 °C were measured through immunohistochemical staining with anti-Pfs25 antibodies as previously described [33,56]. Oocysts and sporozoites were counted as described previously [20,57]. To evaluate the impact of knocking out CTL4 in *A*. *gambiae* adult female mosquitoes, females were divided into 3 cohorts: either maintained on filtered-sterile 10% sucrose solution alone, provided with a single naïve mouse blood meal, or infected with *P*. *berghei*, as detailed in [52]. The life span of these 3 groups (35 females per group) was monitored for 40 d.

### Immunohistochemical staining and confocal microscopy

IFA and confocal microscopy were done essentially according to Dong and colleagues [56] with modifications. *P*. *falciparum*- and *P*. *berghei*-infected mosquito midguts were dissected at approximately 24 hpi in 1% paraformaldehyde, the blood bolus was removed, and the midguts were washed with PBS and fixed in 4% paraformaldehyde for at least overnight. The midguts were then incubated for 1 h with a blocking solution of 10% goat serum (Sigma) and then further incubated overnight with primary antibodies: anti-*A*. *gambiae* CTL4 rabbit polyclonal or anti-*A*. *gambiae* TEP1 rabbit, at 1:400 dilution; anti-*P*. *falciparum* Pfs25 mouse monoclonal (BEI resources, MRA-28), or anti-*P*. *berghei* Pbs28 mouse monoclonal (kindly provided by Professor Sinden, Imperial College London), at 1:500 dilution. After 3 PBS washes, the midguts were incubated for 2 h with secondary antibody: AlexaFluor568 goat anti-rabbit (Life Technologies, A11011) for CTL4 and TEP1, and AlexaFluor 488 goat anti-mouse (Life Technologies, A11029), at 1:500 dilution, for the parasites. Following another 3 washes, the midguts were stained with DAPI, mounted with ProLong Gold antifade reagent, and examined with a Zeiss LSM 710 confocal microscope, collecting 0.2 to 1 mm optical sections. The confocal microscopy settings were kept at similar level across different slides for the comparison purpose, and DAPI staining intensity from each treatment was used for standardization. Stacks of thin optical slices of the samples were projected with Zeiss Zen software, and overlays of different channels were prepared through ImageJ (Fiji). The fluorescent intensities from the colocalization of total TEP1 protein with *P*. *berghei* or *P*. *falciparum* ookinetes in either control or CTL4^null^ midguts were measured using the Image J (Fiji) software. A total of 30 midguts (with at least 3 ookinetes in each gut) were assayed for each treatment. The intensities of the green fluorescence of either *P*. *berghei* or *P*. *falciparum* ookinetes were used as internal references for normalization. Mean ratio of intensities of either control or mutant samples was first obtained by dividing TEP1 intensities (red channel) with intensities of Pbs28 (for *P*. *berghei*, green channel) or Pfs25 (for *P*. *falciparum*, green channel). Fold change of TEP1 mean intensities in CTL4^null^ compared to the controls were calculated by dividing the mean ratio of CTL4^null^ with that of control samples (S3 Table).

### Antibiotic treatment and RNAi-mediated gene silencing

Newly emerged adult female mosquitoes were provided a fresh filtered-sterile 10% sucrose solution containing 75 μg/ml gentamicin sulfate (Sigma) and 100 units-μg/ml of penicillin–streptomycin (Thermo Fisher Scientific) for 3 d, as described in [57], which was replaced 1 d before *P*. *berghei* infection. PCR products were generated from cDNA using gene-specific primers that included a T7 promoter sequence (S1 Table). dsRNA was synthesized, at least 50 females per group were injected with the dsRNA, and the efficiency of the gene silencing for each gene of interest was assessed by qRT-PCR (S1 Table), following the protocol as described in [20].

### Bacterial challenge

For the enumeration of endogenous bacteria from mosquito midguts, non-blood-fed mosquitoes were surface sterilized with ethanol and rinsed with PBS, and their midguts were dissected and homogenized in PBS. Dilutions of this homogenate were plated into LB agar plates and incubated at room temperature for 3 d, after which the bacterial colonies were counted and the number of colony-forming units (CFUs) of culturable bacteria was calculated. Total bacterial load was assessed by qRT-PCR to measure 16s RNA abundance, as in [57]. Mosquito longevity assays were performed as in [52], by intrathoracic injection of 350,000 CFU for the *E*. *coli* suspension, 420,000 CFU for the *S*. *aureus* suspension, or 62,000 CFU for *B*. *subtilis* suspension. Three days after the bacterial injection, the abdomens of the mosquitoes infected with *E*. *coli*, *S*. *aureus*, and the control PBS-injected (20 females per group) were dissected to score for the presence of melanized bacterial clumps.

### Fungal challenge

*Beauvaria bassiana* (*B*. *bassiana* strain 80.2, kindly provided by Professor Silverman, University of Massachusetts Medical School) was cultured, and spores (conidia) used for mosquito challenges were collected as in [9,58]. Freshly prepared spores were used for all experiments. A solution (69 nl) containing 2.15 × 10^6^*B*. *bassiana* spores/ml was injected into adult females (50 females per group) using a nanoinjector. At 12 and 48 h after fungal injection, abdomens were dissected and scored for the presence of live or melanized *B*. *bassiana* spores, and images were captured using a Leica DM 2500 microscope. For the midgut fungal infection, a solution containing 2.15 × 10^8^*B*. *bassiana* spores/ml was added to filtered-sterile 10% sucrose, and newly emerged female adult mosquitoes were allowed to feed on this mixture for 48 h, after which the fungus-supplemented sucrose solution was replaced with regular filtered-sterile 10% sucrose solution. Mosquito midguts were washed with PBS and dissected at 24, 48, and 72 h and 5 and 7 d after fungal challenge, and the presence of live or melanized fungus was evaluated. To measure the longevity following challenge with *B*. *bassiana*, 3- to 4-day-old female mosquitoes were dipped (i.e., surface exposed) for 10 min in a 1-ml solution containing 1 × 10^9^*B*. *bassiana* spores/ml and 0.05% Tween-20 [59]. Dipped females from each group were transferred to cups and kept on filtered-sterile 10% sucrose solution, and dead mosquitoes were counted and removed from cups daily until all females were dead.

### Statistical analysis

All experiments were repeated at least 3 times. Each biological replicate corresponds to a different mosquito population cage, and each population corresponds to a different generation. All graphs were generated using GraphPad Prism8 software, and the statistical methods used for each experiment are indicated in the respective figure legends. Detailed values and statistics are presented in S2 Table. The statistical analysis for *Plasmodium* infection experiments was also performed excluding zeros (noninfected females) (S4 Fig).

## Supporting information

S1 Fig Generation of the *CTL4*-gRNA-expressing transgenic line. The generation of the *CTL4*-gRNA-expressing transgenic line is outlined.
(DOCX)

S2 Fig *Plasmodium* infection intensities do not differ between X1, Cas9, and CTL4-gRNA mosquitoes. *P*. *falciparum* (**a**) and *P*. *berghei* (**b**)infection intensity in X1, Cas9, and CTL4-gRNA *A*. *gambiae* females at 8 or 10 dpi, respectively. No significant differences between the medians (horizontal red lines) of the 3 groups are represented by the horizontal black lines above the graphs.
(DOCX)

S3 Fig Longevity of adult female CTL4^null^ *A*. *gambiae*. Life spans of non-blood-fed (sugar-fed) (**a**), naïve blood-fed (**b**), and *P*. *berghei*-infected blood-fed (**c**) did not differ between control and CTL4^null^ *A*. *gambiae* females (Kaplan–Meier survival analysis with a log-rank test; SE of replicates are indicated).
(DOCX)

S4 Fig *Plasmodium* infection results excluding uninfected individuals. (DOCX)

S1 Table List of gRNA target sequences and primers used in the study. (DOCX)

S2 Table Statistical analysis of *Plasmodium* infections. (DOCX)

S3 Table Quantification of fluorescent intensity from antibody staining of ookinetes and TEP1 in the midgut at 24 hpi in Fig 7G. N indicates total gut samples assayed, with only the values of 20 representatives shown. Mean ratio and standard errors (SE) were calculated from the total parasites.
(DOCX)

S1 File Sequences: Control vs. CTL4^null^. (DOCX)

S2 File CTL4 gene and protein sequences used for antibody production and qPCR. (DOCX)

S3 File Sequences used for polyclonal antibodies production. (DOCX)

S4 File Sequences from the lower gel from qPCR amplification of CTL4^null^ in Fig 1D. (DOCX)

S5 File The underlying data for all graphs. (XLSX)

S1 Raw Images The original PCR gels of Fig 1C and 1D and western blots of Fig 1E. (PDF)

## Abbreviations

CFU: colony-forming unit
dsRNA: double-stranded RNA
gRNA: guide RNA
hpi: hours postinfection
Imd: immune deficiency
kd: knockdown
LB: Luria broth
PBM: post-blood meal
qRT-PCR: quantitative reverse transcription-PCR
RNAi: RNA interference
