# Supplementary material for: C-type lectin 4 regulates broad-spectrum melanization-based refractoriness to malaria parasites
Source: PLoS Biol. 2022 Jan 13;20(1):e3001515. doi: 10.1371/journal.pbio.3001515 (PMC8791531; doi:10.1371/journal.pbio.3001515)
Supplement: S1 Fig — The generation of the CTL4-gRNA-expressing transgenic line is outlined. (DOCX) [file pbio.3001515.s001.docx]

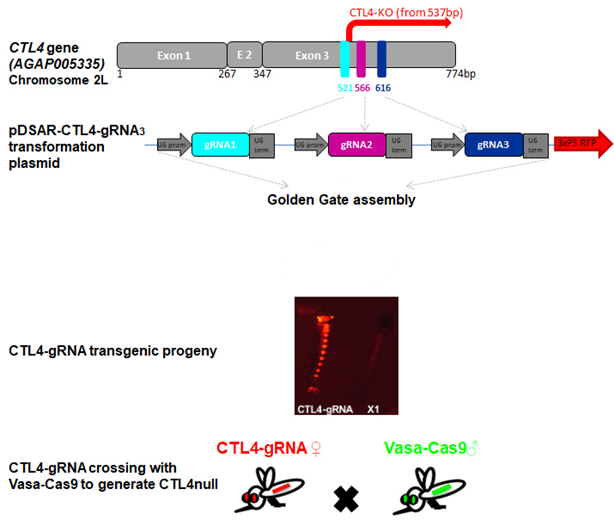


**S1 Fig. Generation of the *CTL4-*gRNA*-*expressing transgenic line.** The generation of the *CTL4*-gRNA*-*expressing transgenic line is outlined.
