# Supplementary material for: C-type lectin 4 regulates broad-spectrum melanization-based refractoriness to malaria parasites
Source: PLoS Biol. 2022 Jan 13;20(1):e3001515. doi: 10.1371/journal.pbio.3001515 (PMC8791531; doi:10.1371/journal.pbio.3001515)
Supplement: S2 Fig — P. falciparum (a) and P. berghei (b)infection intensity in X1, Cas9, and CTL4-gRNA A. gambiae females at 8 or 10 dpi, respectively. No significant differences between the medians (horizontal red lines) of the 3 groups are represented by the horizontal black lines above the graphs. (DOCX) [file pbio.3001515.s002.docx]

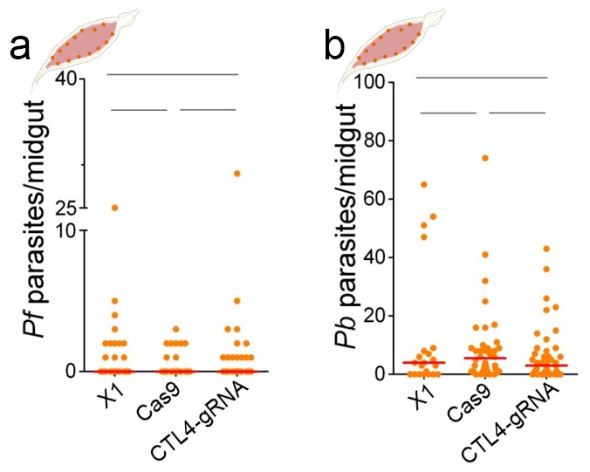


**S2 Fig. *Plasmodium* infection intensities do not differ between X1, Cas9 and CTL4-gRNA mosquitoes.** *P. falciparum* (**a**) and *P. berghei* (**b**) infection intensity in X1, Cas9 and CTL4-gRNA *A. gambiae* females at 8 or 10 dpi, respectively. No significant differences between the medians (horizontal red lines) of the three groups are represented by the horizontal black lines above the graphs.
