# Supplementary material for: C-type lectin 4 regulates broad-spectrum melanization-based refractoriness to malaria parasites
Source: PLoS Biol. 2022 Jan 13;20(1):e3001515. doi: 10.1371/journal.pbio.3001515 (PMC8791531; doi:10.1371/journal.pbio.3001515)
Supplement: S3 Fig — Life spans of non-blood-fed (sugar-fed) (a), naïve blood-fed (b), and P. berghei-infected blood-fed (c) did not differ between control and CTL4null A. gambiae females (Kaplan–Meier survival analysis with a log-rank test; SE of replicates are indicated). (DOCX) [file pbio.3001515.s003.docx]

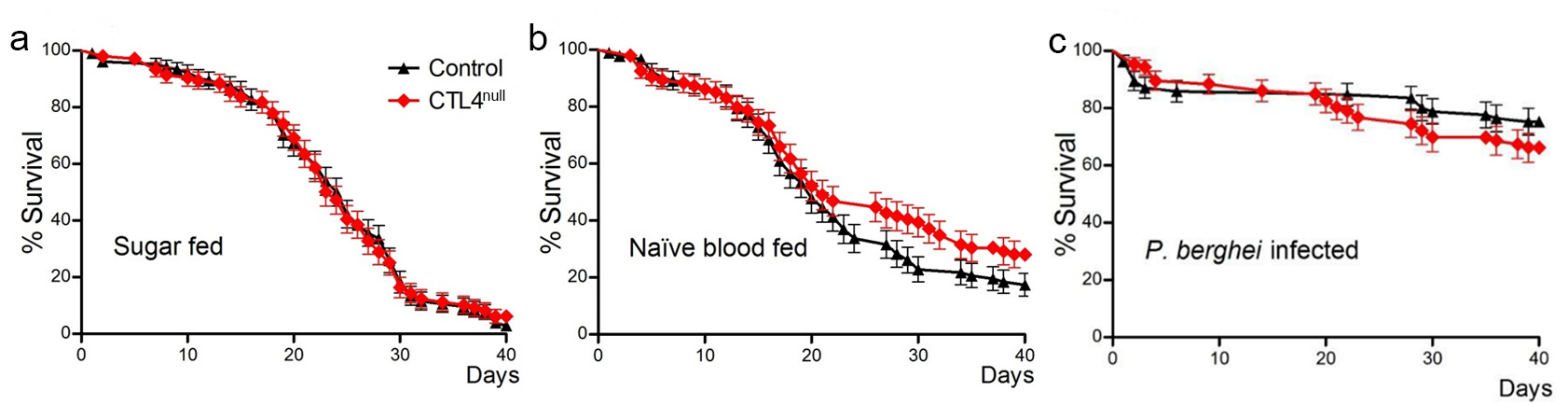


**S3 Fig. Longevity of adult female CTL4^null^ *A. gambiae*.** Life spans of non-blood-fed (sugar-fed) (**a**), naïve blood-fed (**b**) and *P. berghei-*infected blood-fed (**c**) did not differ between control and CTL4^null^ *A. gambiae* females (Kaplan-Meier survival analysis with a log-rank test; SE of replicates are indicated).
