# Supplementary figures and images for: C-type lectin 4 regulates broad-spectrum melanization-based refractoriness to malaria parasites
Source: PLoS Biol. 2022 Jan 13;20(1):e3001515. doi: 10.1371/journal.pbio.3001515 (PMC8791531; doi:10.1371/journal.pbio.3001515)

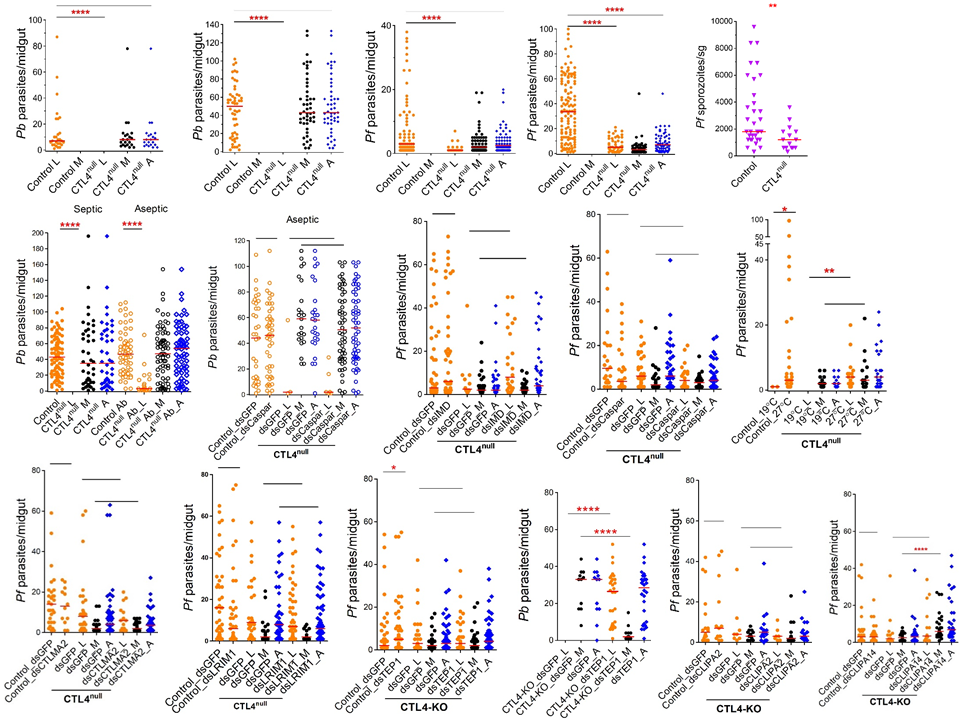


**S4 Fig. *Plasmodium* infection data excluding uninfected individuals.**

Supplement: S4 Fig — (DOCX) [file pbio.3001515.s004.docx]

**S2 File. CTL4 gene and protein sequences used for antibody production and qPCR.**

**
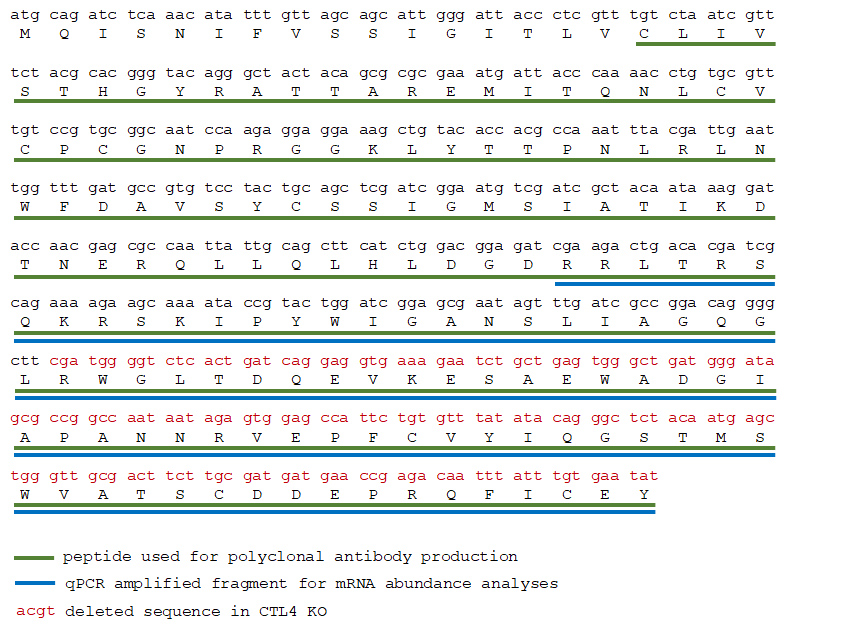
**

Supplement: S2 File — (DOCX) [file pbio.3001515.s009.docx]

Figure 1C gel

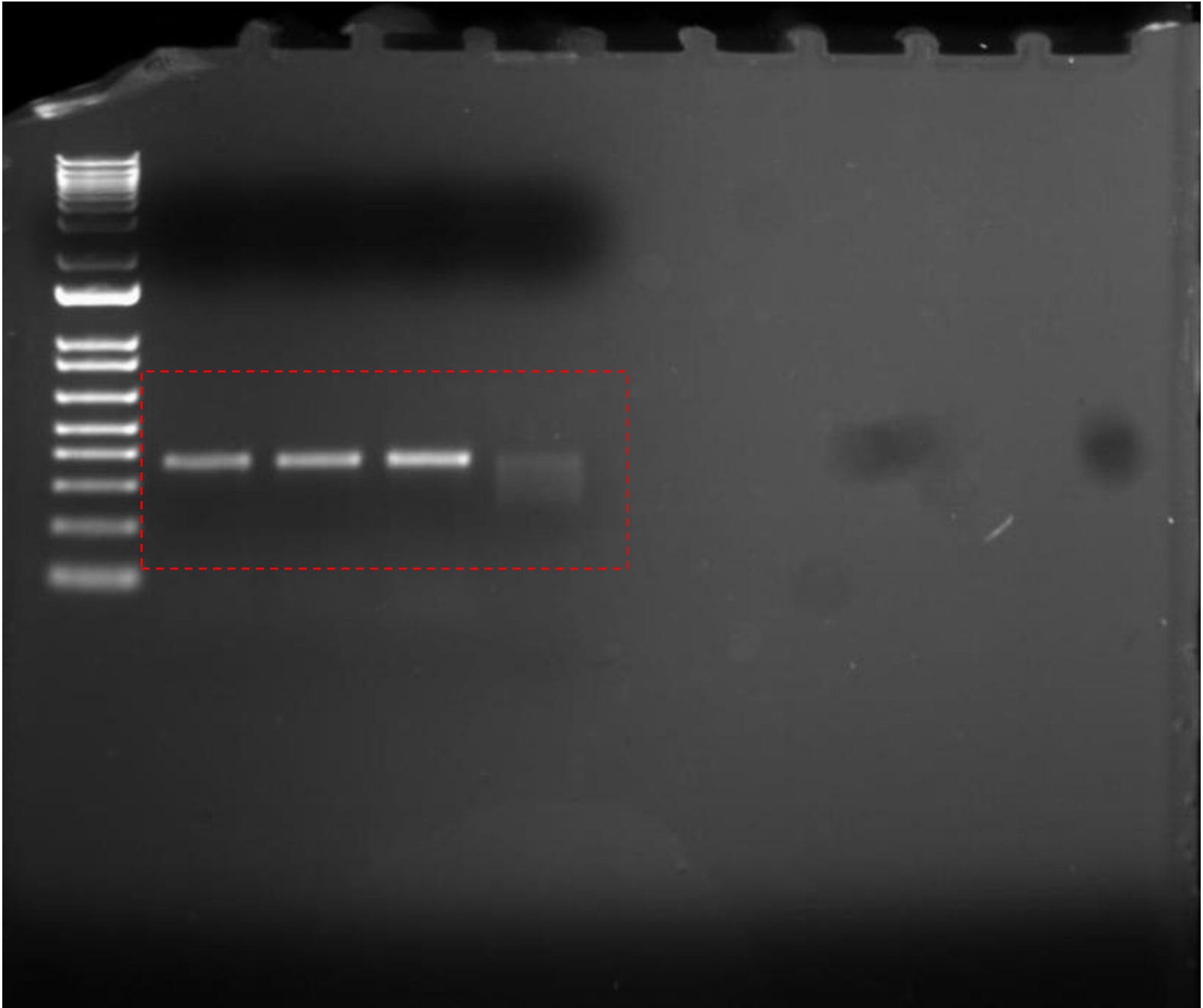

Figure 1D gel

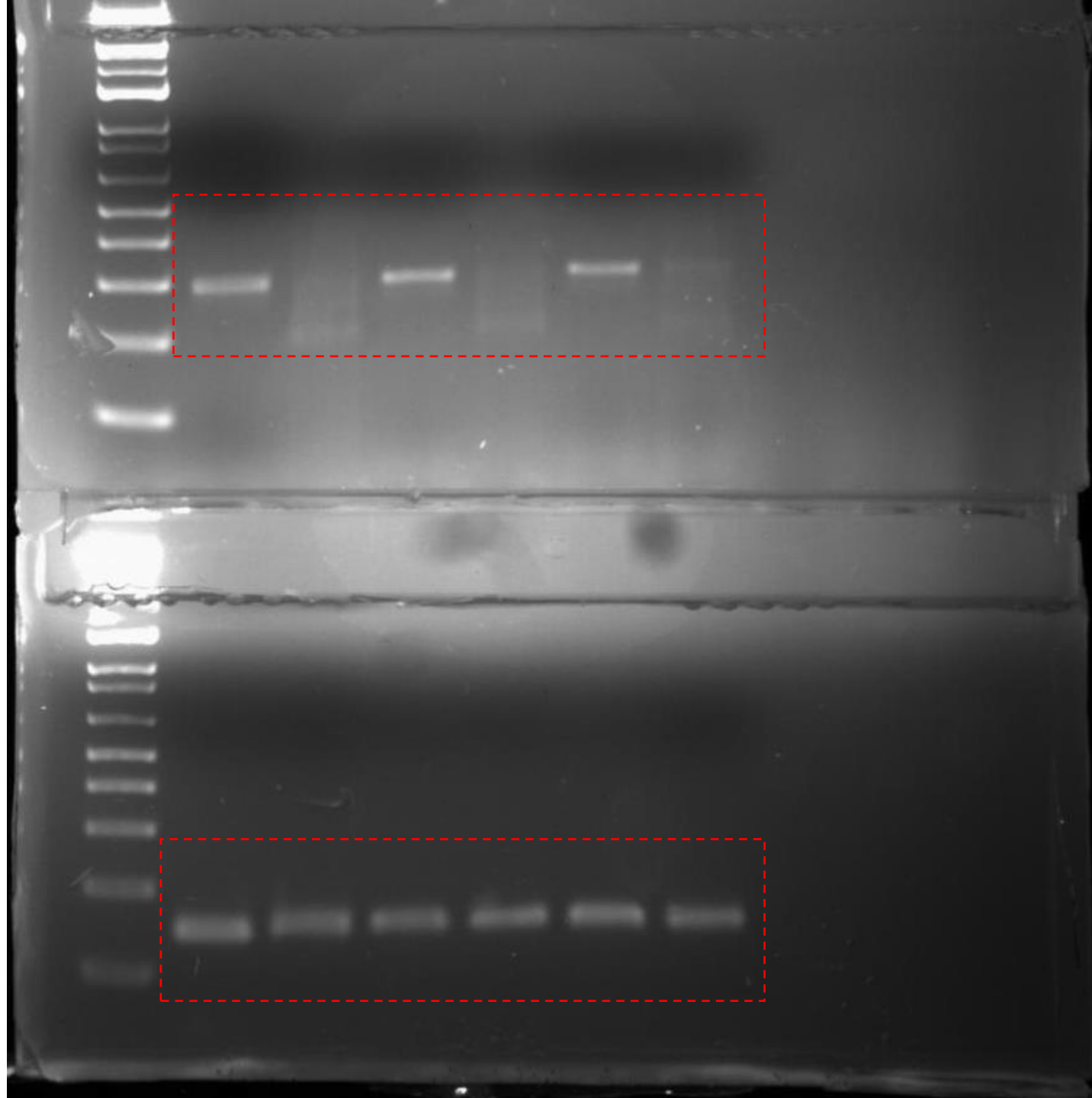

Figure 1E blot

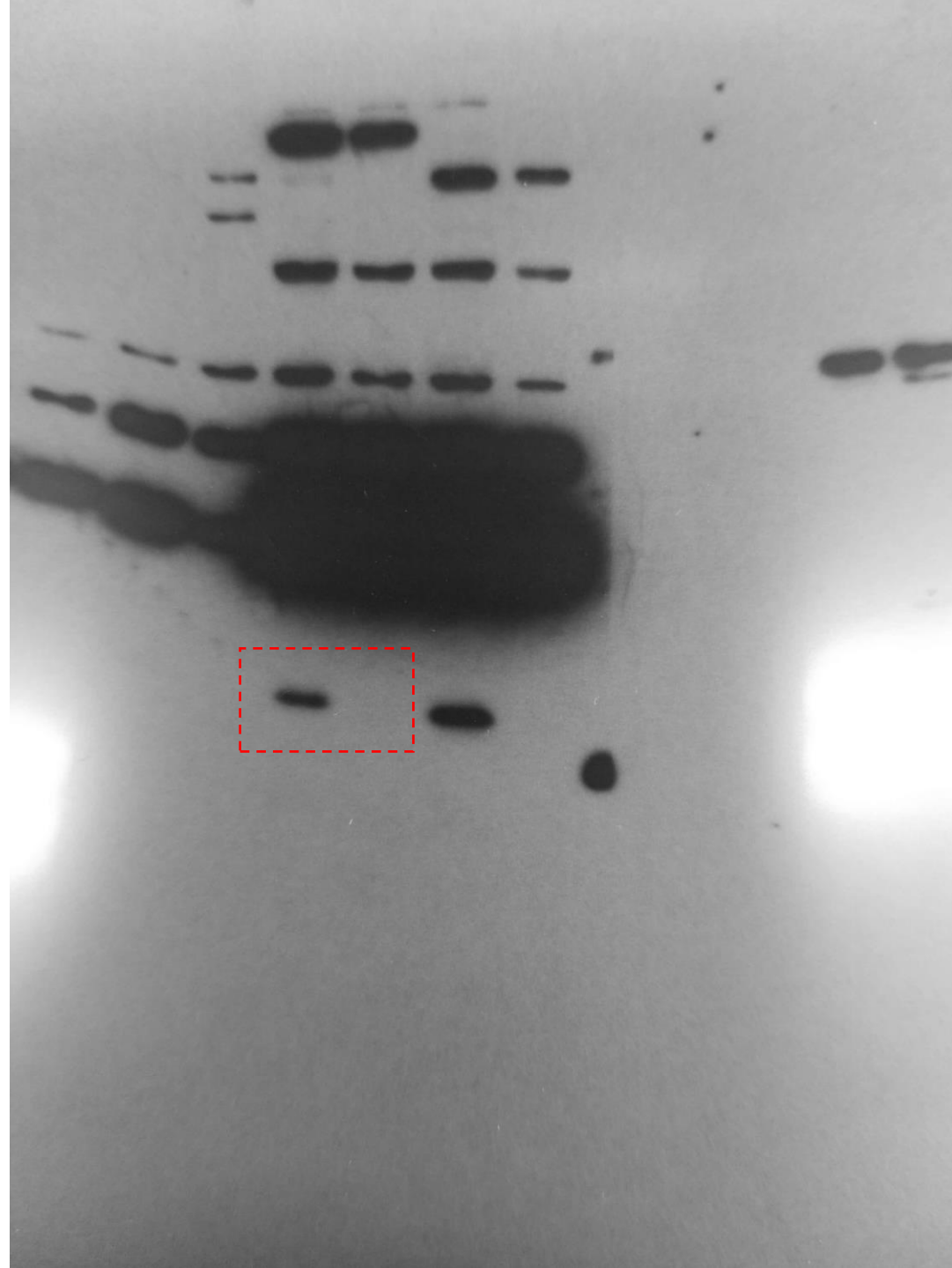

Figure 1E blot

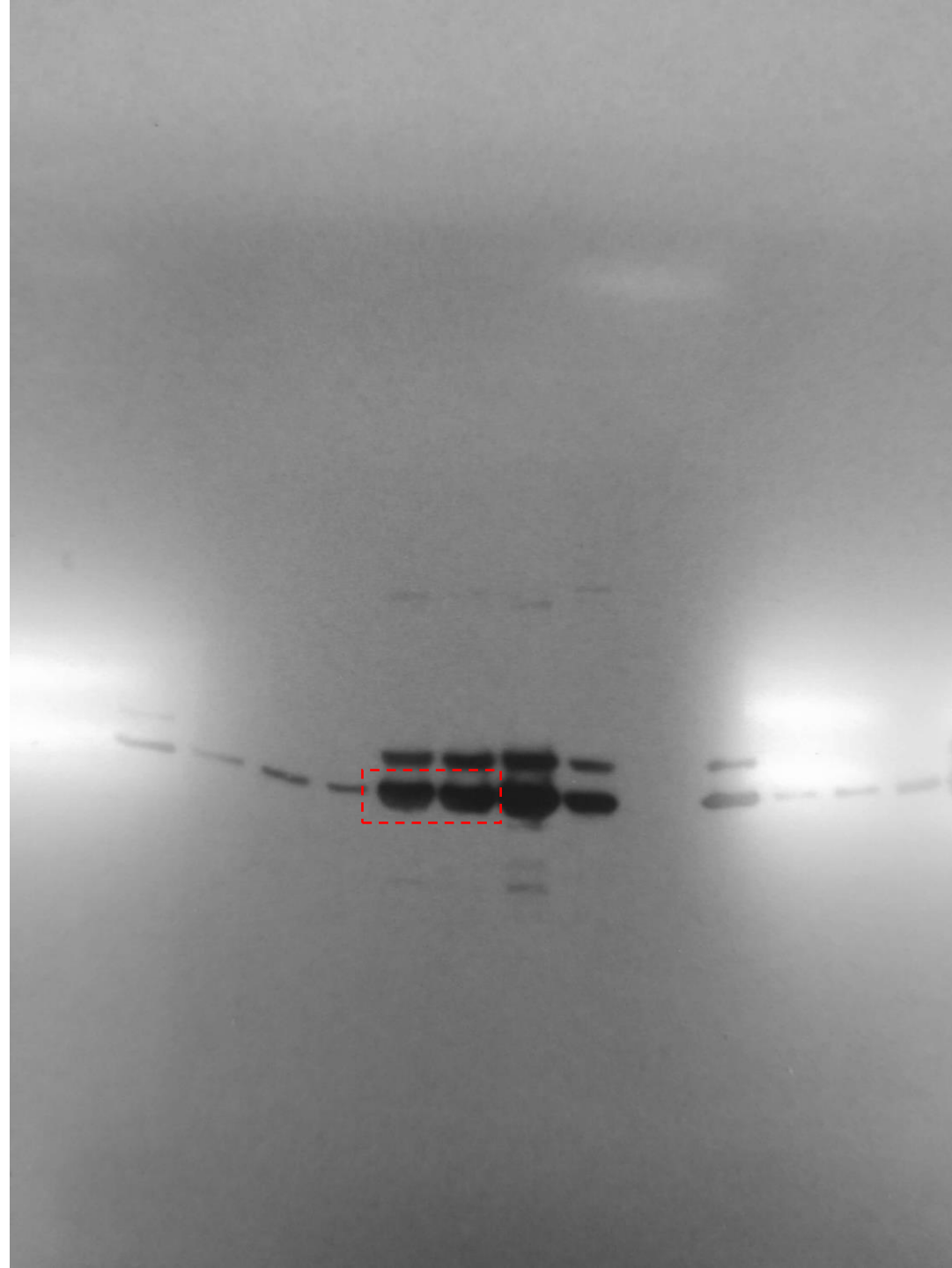

Supplement: S1 Raw Images — (PDF) [file pbio.3001515.s013.pdf]
