# Supplementary material for: C-type lectin 4 regulates broad-spectrum melanization-based refractoriness to malaria parasites
Source: PLoS Biol. 2022 Jan 13;20(1):e3001515. doi: 10.1371/journal.pbio.3001515 (PMC8791531; doi:10.1371/journal.pbio.3001515)
Supplement: S1 Table — (DOCX) [file pbio.3001515.s005.docx]

**S1 Table. List of gRNA target sequences and primers used in the study.**

**gRNA target sequences:**

| **Name** | **Sequence (with PAM)** | **Cleavage Position (%)*** | **Direction*** | **GC Contents (%, w/o PAM)*** | **Out-of-frame Score*** | **Mismatches*** | | | **Genomic location^#^** | **Exon^#^** | **Self-complementarity^#^** | **Off-targets^#^** | | | | **Efficiency^#^** |
| --- | --- | --- | --- | --- | --- | --- | --- | --- | --- | --- | --- | --- | --- | --- | --- | --- |
|  |  |  |  |  |  | **0** | **1** | **2** |  |  |  | **0** | **1** | **2** | **3** |  |
| gRNA1 | GCCGGACAGGGGCTTCGATGGGG | 72.0 | + | 70 | [66](javascript:%20void(0);) | [1](javascript:%20void(0);) | 0 | 0 | 2L:14233634 | 3 | 1 | 0 | 0 | 0 | 0 | 60.32 |
| gRNA2 | GAATCTGCTGAGTGGGCTGATGG | 77.4 | + | 55 | 58.8 | [1](javascript:%20void(0);) | 0 | 0 | 2L:14233589 | 3 | 1 | 0 | 0 | 0 | 0 | 48.58 |
| gRNA3 | GCCATTCTGTGTTTATATAC  AGG | 83.3 | + | 35 | [68.7](javascript:%20void(0);) | [1](javascript:%20void(0);) | 0 | 0 | 2L:14233539 | 3 | 1 | 0 | 0 | 0 | 0 | 36.73 |

*: <http://www.rgenome.net/cas-designer/> ^#^: <http://chopchop.cbu.uib.no/>

**Primers for generation of construct and verification of gene knockout:**

| **Primer name** | **Purpose** | **Sequence fwd** | **Sequence rev** |
| --- | --- | --- | --- |
| gRNACTL4_1 | cloning of gRNA1 target DNA sequences to pKSB-gRNA-1 | ccttGCCGGACAGGGGCTTCGATG | aaacCATCGAAGCCCCTGTCCGGC |
| gRNACTL4_2 | cloning of gRNA2 target DNA sequences to pKSB-gRNA-2 | ccttGAATCTGCTGAGTGGGCTGA | aaacTCAGCCCACTCAGCAGATTC |
| gRNACTL4_3 | cloning of gRNA3 target DNA sequences to pKSB-gRNA-3 | ccttGCCATTCTGTGTTTATATAC | aaacGTATATAAACACAGAATGGC |
| CTL4-KO-flanking | PCR primers for confirmation of CTL4 knockout | GGATACCAACGAGCGCCAAT | GCGTTTTCTCGGCAGAAACAT |

**Primers for RNAi-mediated gene silencing (T7 promoter sequence underlined):**

| **Gene name** | **Accession number** | **Sequence fwd** | **Sequence rev** | **Gene kd**  **%** | **Reference** |
| --- | --- | --- | --- | --- | --- |
| *AgCaspar* | AGAP006473 | TAATACGACTCACTATAGCCGCTTTTCTAAACGCTGTC | TAATACGACTCACTATAGAAACAGGTTGCATGTGTGGA | 63.25 | 23 |
| *AgCLIPA2* | AGAP011790 | TAATACGACTCACTATAGGGATCCTAACAACGGCACACTGTGTGA | TAATACGACTCACTATAGGGTCCTGATCGCCATGATTGGTGGTGCT | 87.45 | 30 |
| *AgCLIPA14* | AGAP011788 | TAATACGACTCACTATAGGGCGGCATCATCGACATCCGTGTC | TAATACGACTCACTATAGGGGTTGCTGTCGGCGACACGCTCCT | 91.04 | 30 |
| *AgCTLMA2* | AGAP005334 | TAATACGACTCACTATAGGGCAAACCGTTCGAGGAGAAAG | TAATACGACTCACTATAGGGTCTGCTGCGTACATTGGAAA | 87.02 | 20 |
| *AgIMD* | AGAP004959 | TAATACGACTCACTATAGGGGAATTTCCCAAATGGTGTG | TAATACGACTCACTATAGGGTGTGTAGATTGCTCGCGTTC | 68.10 | 35 |
| *AgLRIM1* | AGAP006348 | TAATACGACTCACTATAGGGCCAGAATGA | TAATACGACTCACTATAGGCAGCTCGATC | 80.96 | 20 |
| *AgTEP1* | AGAP010815 | TAATACGACTCACTATAGGGTTTGTGGGCCTTAAAGCGCTG | TAATACGACTCACTATAGGGACCACGTAACCGCTCGGTAAG | 79.32 | 7,23,30,32 |
| *GFP* |  | TAATACGACTCACTATAGGGTTCATCTGCACCACCGGC | TAATACGACTCACTATAGGGCTGGTAGTGGTCGGCGAG |  | 20 |

**Primers for qRT-PCR analysis:**

| **Gene name** | **Accession number** | **Sequence fwd** | **Sequence rev** | **Reference** |
| --- | --- | --- | --- | --- |
| *AgCTL4* | AGAP005335 | ATCGGAATGTCGATCGCTAC | TTCATCATCGCAAGAAGTCG | 20 |
| *AgCaspar* | AGAP006473 | AACACTGAACCACGAAAGCA | GAGCTCGGTTCACGGATG | new |
| *AgCLIPA2* | AGAP011790 | GATACTACCTGCACGGGTTGGT | CAGTATAAGGTATCTGCTTCTGATGGC | 29 |
| *AgCLIPA14* | AGAP011788 | AGAACGTGTTTGGCAAGGAG | GTGCTGCGCAGTGACTGTT | new |
| *AgCTLMA2* | AGAP005334 | CACAGTGGTTCGTGGTGACCTA | CATGGGTTTTGTTGAAGAATATCATC | 20 |
| *AgIMD* | AGAP004959 | CGAGACTATGGCTCACACCA | TGTGTAGATTGCTCGCGTTC | 35 |
| *AgLRIM1* | AGAP006348 | CGCAACAACAAACTGGTACTG | AAATCACGCAACGTTCCAC | new |
| *AgTEP1* | AGAP010815 | AAAGCTGTTGCGTCAGGG | TTCTCCCACACACCAAACGAA | 7 |
| *AgS7* | AGAP010592 | AGAACCAGCAGACCACCATC | GCTGCAAACTTCGGCTATTC | 20 |
