# Supplementary material for: C-type lectin 4 regulates broad-spectrum melanization-based refractoriness to malaria parasites
Source: PLoS Biol. 2022 Jan 13;20(1):e3001515. doi: 10.1371/journal.pbio.3001515 (PMC8791531; doi:10.1371/journal.pbio.3001515)
Supplement: S2 Table — (DOCX) [file pbio.3001515.s006.docx]

**S2 Table. Statistical analysis of *Plasmodium* infections.**

| **Time**  **PBM** | **Parasite**  **Species** | **Treatment**  **dsRNA,^o^C** | **Control**  **Infection Intensity** | | **CTL4^null^**  **Infection Intensity** | | **Intensity**  **Mann-Whitney** | | **Prevalence**  **% Fisher** | | **Melanized in All**  **%** | |
| --- | --- | --- | --- | --- | --- | --- | --- | --- | --- | --- | --- | --- |
|  |  |  | **N, Median** | **Range** | **N, Median** | **Range** |  | | **Control** | **CTL4^null^** | **Control** | **CTL4^null^** |
| 10 dpi | Pb |  | 35, 6 | 0-87 | 21, 0 | 0-0 | <0.0001 (low) | | 86 | 0, <0.0001 | 0 | 100 |
| 10 dpi | Pb |  | 56, 45 | 0-102 | 50, 0 | 0-0 | <0.0001 (high) | | 93 | 0, <0.0001 | 0 | 100 |
| 8 dpi | Pf NF54 |  | 116, 1 | 0-38 | 85, 0 | 0-7 | <0.0001 (low) | | 60 | 19, <0.0001 | 0 | 87 |
| 8 dpi | Pf NF54 |  | 128, 32 | 0-100 | 72, 2 | 0-21 | <0.0001 (high) | | 97 | 63, <0.0001 | 0 | 42 |
| 14 dpi | Pf NF54 |  | 45, 1,500 | 0-10,000 | 47, 0 | 0-3,600 | <0.0001 (low) | | 73 | 32, <0.0001 | - | - |
| 19 hpi | Pb |  | 35, 536 | 120-1,444 | 25, 488 | 100-1,500 | ns | | - | - | - | - |
| 19 hpi | Pf NF54 |  | 51, 320 | 12-1,080 | 27, 268 | 40-716 | ns | | - | - | - | - |
| 10 dpi | Pb |  | 93, 42 | 0-104 | 44, 0 | 0-0 | <0.0001 | | - | - | 0 | 100 |
| 10 dpi | Pb (aseptic) |  | 68, 47 | 3-112 | 70, 0 | 0-71 | <0.0001 | | - | - | 0 | 94 |
|  |  |  |  |  |  |  | **Control** | **CTL4^null^** |  |  |  |  |
| 10 dpi | Pb (aseptic) | GFP | 35, 44 | 1-109 | 25, 0 | 0-58 | - | - | 100 | 12 | 0 | 96 |
| 10 dpi | Pb (aseptic) | Caspar | 46, 46 | 0-112 | 55, 0 | 0-29 | ns | ns | 98, ns | 11, ns | 0 | 98 |
| 24 hpi | Pf lumen (ookinetes) | 19^o^C | 24, 12 | 0-54 | - | - | - | - | - | - | - | - |
| 40 hpi | Pf lumen (ookinetes) | 19^o^C | 24, 74 | 16-216 | - | - | <0.0001 | - | ns | - | - | - |
| 24 hpi | Pf lumen (ookinetes) | 27^o^C | 24, 166 | 8-500 | - | - | 0.0008 | - | <0.0001 | - | - | - |
| 13 dpi | Pf gut oocysts | 19^o^C | 39, 40 | 0-102 | - | - | - | - | - | - | - | - |
| 8 dpi | Pf gut oocysts | 27^o^C | 40, 95 | 3-178 | - | - | <0.0001 | - | <0.0001 | - | - | - |
| 24 hpi | Pf epithelium ookinetes | 19^o^C | 45, 4 | 0-10 | - | - | - | - | - | - | - | - |
| 24 hpi | Pf epithelium ookinetes | 27^o^C | 50, 39 | 6-218 | - | - | <0.0001 | - | 0.0032 | - | - | - |
| 40 hpi | Pf epithelium ookinetes | 19^o^C | 57, 5 | 0-19 | - | - | - | - | - | - | - | - |
| 40 hpi | Pf epithelium early oocysts | 27^o^C | 56, 46 | 2-228 | - | - | <0.0001 | - | 0.0007 | - | - | - |
| 13 dpi | Pf gut oocysts | 19^o^C | 40, 4 | 0-44 | - | - | - | - | - | - | - | - |
| 8 dpi | Pf gut oocysts | 27^o^C | 47, 48 | 1-112 | - | - | <0.0001 | - | <0.0001 | - | - | - |
| 8 dpi | Pf NF54 | 19^o^C | 55, 0 | 0-1 | 35, 0 | 0-0 | - | - | 4 | 0 | 0 | 100 |
| 8 dpi | Pf NF54 | 27^o^C | 54, 1 | 0-97 | 35, 0 | 0-20 | <0.0001 | <0.0001 | 54, <0.0001 | 49, <0.0001 | 0 | 47 |
| 8 dpi | Pf NF54 | GFP | 112, 0 | 0-65 | 60, 0 | 0-9 | - | - | 44 | 20 | 0 | 62 |
| 8 dpi | Pf NF54 | IMD | 85, 1 | 0-73 | 66, 0 | 0-45 | 0.0475 | 0.0219 | 55, ns | 37, 0.0497 | 0 | 21 |
| 8 dpi | Pf NF54 | GFP | 27, 6 | 0-63 | 60, 1 | 0-31 | - | - | 89 | 55 | 0 | 33 |
| 8 dpi | Pf NF54 | Caspar | 47, 1 | 0-39 | 43, 0 | 0-20 | 0.0011 | ns | 64, 0.0087 | 47, ns | 0 | 49 |
| 8 dpi | Pf NF54 | GFP | 36, 10.5 | 0-59 | 62, 0 | 0-60 | - | - | 81 | 47 | 0 | 21 |
| 8 dpi | Pf NF54 | CTLMA2 | 15, 7 | 0-26 | 54, 0 | 0-20 | ns | 0.0248 | 80, ns | 28, ns 0.0545 | 0 | 45 |
| 8 dpi | Pf NF54 | GFP | 55, 6 | 0-65 | 54, 1 | 0-57 | - | - | 78 | 54 | 0 | 23 |
| 8 dpi | Pf NF54 | LRIM1 | 47, 3 | 0-75 | 77, 2 | 0-55 | ns | ns | 83, ns | 65, ns | 0 | 15 |
| 8 dpi | Pf NF54 | GFP | 85, 1 | 0-54 | 71, 0 | 0-42 | - | - | 59 | 41 | 0 | 40 |
| 8 dpi | Pf NF54 | TEP1 | 66, 3 | 0-55 | 76, 0 | 0-37 | 0.0018 | ns | 76, 0.0373 | 41, ns | 0 | 40 |
| 8 dpi | Pf NF54 | GFP | 28, 1.5 | 0-42 | 21, 0 | 0-36 | - | - | 64 | 38 | 0 | 71 |
| 8 dpi | Pf NF54 | CLIPA2 | 32, 0 | 0-45 | 22, 0 | 0-11 | ns | ns | 41, ns | 32, ns | 0 | 82 |
| 8 dpi | Pf NF54 | GFP | 55, 1.5 | 0-42 | 47, 0 | 0-36 | - | - | 60 | 29 | 0 | 64 |
| 8 dpi | Pf NF54 | CLIPA14 | 46, 2 | 0-23 | 34, 0 | 0-34 | ns | ns | 69, ns | 41, ns | 0 | 82 |
| 10 dpi | Pb | GFP | - | - | 12, 0 | 0-0 | - | - | - | 0 | - | 100 |
| 10 dpi | Pb | TEP1 | - | - | 30, 26.5 | 1-52 | - | <0.0001 | - | 100 | - | 6 |
