# Supplementary material for: C-type lectin 4 regulates broad-spectrum melanization-based refractoriness to malaria parasites
Source: PLoS Biol. 2022 Jan 13;20(1):e3001515. doi: 10.1371/journal.pbio.3001515 (PMC8791531; doi:10.1371/journal.pbio.3001515)
Supplement: S3 Table — N indicates total gut samples assayed, with only the values of 20 representatives shown. Mean ratio and standard errors (SE) were calculated from the total parasites. (DOCX) [file pbio.3001515.s007.docx]

**S3 Table. Quantification of fluorescent intensity from antibody staining of ookinetes and TEP1 in the midgut at 24 hpi in Figure 7G.**


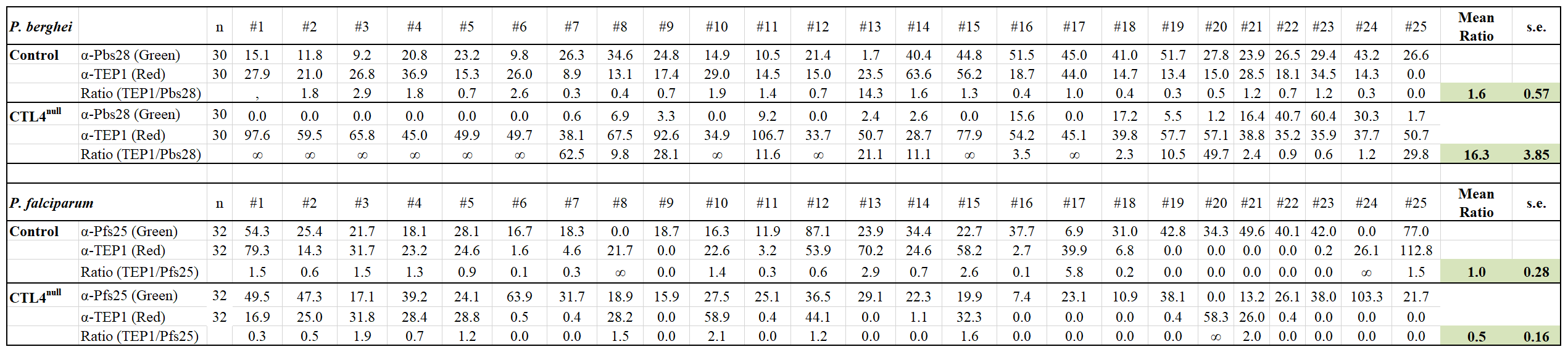


∞ indicates where there is no staining of the parasite with either Pbs28 or Pfs25 antibodies.
