# Supplementary material for: C-type lectin 4 regulates broad-spectrum melanization-based refractoriness to malaria parasites
Source: PLoS Biol. 2022 Jan 13;20(1):e3001515. doi: 10.1371/journal.pbio.3001515 (PMC8791531; doi:10.1371/journal.pbio.3001515)
Supplement: S1 File — (DOCX) [file pbio.3001515.s008.docx]

**S1 File. Sequences: Control vs CTL4 ^null^.**

Primer Rev CTL4 flanking: GCGTTTTCTCGGCAGAAACAT (Reverse complement: ATGTTTCTGCCGAGAAAACGC)

gRNA1: GCCGGACAGGGGCTTCGATG

gRNA2: GAATCTGCTGAGTGGGCTGA

gRNA3: GCCATTCTGTGTTTATATAC

**>X1-control (PCR fragment from X1 wt *A. gambiae*).** Identical to PCR fragments from Vasa-Cas-9 and CTL4-gRNA.

CGAAGACTGACACGATCGCAGAAAAGAAGCAAAATACCGTACTGGATCGGAGCGAATAGTTTGATCGCCGGACAGGGGCTTCGATGGGGTCTCACTGATCAGGAGGTGAAAGAATCTGCTGAGTGGGCTGATGGGATAGCGCCGGCCAATAATAGAGTGGAGCCATTCTGTGTTTATATACAGGGCTCTACAATGAGCTGGGTTGCGACTTCTTGTGATGATGAACCGAGACAATTTATTTGTGAATATTAGAAGATTAATGGGACCAATGCATAGCATTGAAAATAAACTCCTCGAATGTTTCTGCCGAGAAAACGCA

**>CTL4^null^ (PCR fragment from CTL4-knockout (CTL4-KO) *A. gambiae*)**

TCGAAGACTGACACGATCGCAGAAAAGAAGCAAAATACCGTACTGGATCGGAGCGAATAGTTTGATCGCCGGACAGGGGCTTCCCTCCCCCCGGGGGGGGGAAATTTGTTTTGTTGTTTTGGGACGACATACCTTTTTTTTTTTTTAAAGAATAGGGGCGGGGGCCTGGGACCGATGAATAAAATTTCTAATGTTGTTCTGGAAAGAAAAAAAAAAAAAAAAAAAATATTTTTTTTTATTATTTGGTCATAGGAAAAAAACCAAAACATTGAAATTAAACTCCTCCTATGGTTCTGCCGAGAAAACGAA

**>Sequencing results Control vs CTL4^null^**


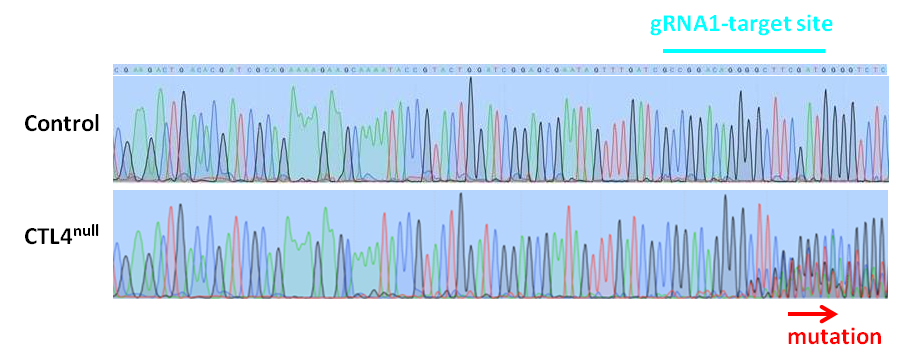


**> Control DNA-protein alignment**

cga aga ctg aca cga tcg cag aaa aga agc aaa ata ccg tac tgg atc gga gcg aat agt ttg atc gcc gga cag ggg ctt cga tgg ggt 
  R    R    L   T    R   S    Q    K    R    S    K    I    P   Y   W   I    G    A    N   S   L I  A G Q G L R W G ctc act gat cag gag gtg aaa gaa tct gct gag tgg gct gat ggg ata gcg ccg gcc aat aat aga gtg gag cca ttc tgt gtt tat ata 
  L   T   D   Q    E   V  K    E   S   A E   W   A   D   G   I   A    P    A   N   N   R   V    E    P   F   C  V  Y  I  
cag ggc tct aca atg agc tgg gtt gcg act tct tgt gat gat gaa ccg aga caa ttt att tgt gaa tat
  Q   G    S   T   M S W V A T S C D D E P R Q F I C E Y

**> CTL4^null^ DNA-protein alignment**

cga aga ctg aca cga tcg cag aaa aga agc aaa ata ccg tac tgg atc gga gcg aat agt ttg atc gcc gga cag ggg ctt ccc tcc ccc
  R    R    L   T    R   S    Q    K    R    S    K    I    P   Y   W   I    G    A    N   S   L I  A G Q G L P S P cgg ggg ggg gaa att tgt ttt gtt gtt ttg gga cga cat acc ttt ttt ttt ttt taa 
 R G G E I C F V V L G   R   H    T   F  F  F  F   *

**>DNA alignment Control vs CTL4^null^**

Control 1 CGAAGACTGACACGATCGCAGAAAAGAAGCAAAATACCGTACTGGATCGG 50

||||||||||||||||||||||||||||||||||||||||||||||||||

CTL4-KO 1 CGAAGACTGACACGATCGCAGAAAAGAAGCAAAATACCGTACTGGATCGG 50

Control 51 AGCGAATAGTTTGATCGCCGGACAGGGGCTTCGAT-------GGGGTCTC 93

||||||||||||||||||||||||||||||||..| ||||

CTL4-KO 51 AGCGAATAGTTTGATCGCCGGACAGGGGCTTCCCTCCCCCCGGGGG---- 96

Control 94 ACTGATCAGGAGGTGAAAGAATCTGCTGAGTGGGCTGATGGGATAGCGCC 143

||.|| |||.||.|..||.| |..||||| ||.|

CTL4-KO 97 -----------GGGGA---AATTTGTTTTGTTG--TTTTGGGA---CGAC 127

Control 144 GGCCAATAATAGAGTGGAGCCATTCTGTGTTTATA---TACAGGGCTCTA 190

||| ||.||.|.|.|||.|| .|.||||

CTL4-KO 128 -----ATA-----------CCTTTTTTTTTTTTTAAAGAATAGGG----- 156

Control 191 CAATGAGCTGGG--TTGCGAC-------------TTCTTGTGATGAT--- 222

||.||| .||.||| ||||..||.||.|

CTL4-KO 157 ------GCGGGGGCCTGGGACCGATGAATAAAATTTCTAATGTTGTTCTG 200

Control 223 GAACCGAGACA------------------ATTTATTTGTGAATATTAGAA 254

||| |||.| |||| |||.|.|.||||.|

CTL4-KO 201 GAA---AGAAAAAAAAAAAAAAAAAAAATATTT-TTTTTTATTATTTG-- 244

Control 255 GATTAATGGG------ACCAATGCATAGCATTGAAAATAAACTCCTCGAA 298

.|.||.|| ||||| |.||||||||.||||||||||..|

CTL4-KO 245 --GTCATAGGAAAAAAACCAA-----AACATTGAAATTAAACTCCTCCTA 287

Control 299 TGTTTCTGCCGAGAAAACGCA 319

||.||||||||||||||||.|

CTL4-KO 288 TGGTTCTGCCGAGAAAACGAA 308

**>Protein alignment Control vs CTL4^null^**

**mutation**
