# Supplementary material for: C-type lectin 4 regulates broad-spectrum melanization-based refractoriness to malaria parasites
Source: PLoS Biol. 2022 Jan 13;20(1):e3001515. doi: 10.1371/journal.pbio.3001515 (PMC8791531; doi:10.1371/journal.pbio.3001515)
Supplement: S3 File — (DOCX) [file pbio.3001515.s010.docx]

**S3 File. Sequences used for polyclonal antibodies production.**

**>*Anopheles gambiae* CTL4**

CLIVSTHGYRATTAREMITQNLCVCPCGNPRGGKLYTTPNLRLNWFDAVSYCSSIGMSIATIKDTNERQLLQLHLDGDRRLTRSQKRSKIPYWIGANSLIAGQGLRWGLTDQEVKESAEWADGIAPANNRVEPFCVYIQGSTMSWVATSCDDEPRQFICEY

**>*Anopheles gambiae* TEP1**

QDTFVGLKALTKMAEKISPSRNDYTVQLKYKKSAKYFKINSEQIDVENFVDIPEDTKKLEINVGGIGFGLLEVVYQFNLNLVNFENRFQLDLEKQNTGSDYELRLKVCASYIPQLTDRRSNMALIEVTLPSGYVVDRNPISEQTKVNPIQKTEIRYGGTSVVLYYDNMGSERNCFTLTAYRRFKVALKRPAYVVVYDYYNTNLNAIKVYEVDKQNLCEICDEEDCPAECKK
