# Supplementary material for: C-type lectin 4 regulates broad-spectrum melanization-based refractoriness to malaria parasites
Source: PLoS Biol. 2022 Jan 13;20(1):e3001515. doi: 10.1371/journal.pbio.3001515 (PMC8791531; doi:10.1371/journal.pbio.3001515)

**S4 File. Sequences from the lower gel from qPCR amplification of CTL4^null^ in Figure 1D.**

**> CTL4 sequencing (lane 2)**

CGAAGACTGACACGATCGCAGAAAAGAAGCAAAATACCGTACTGGATCGGAGCGAATAGTTTGATCGCCGGACAGGGGCTCCGCCCTCANCCGGGTGGGGAGTTGCGGGGTGAATTGTTTCAAACCTNCNCATTTGGNTGNNNNNAAANACCCNCN


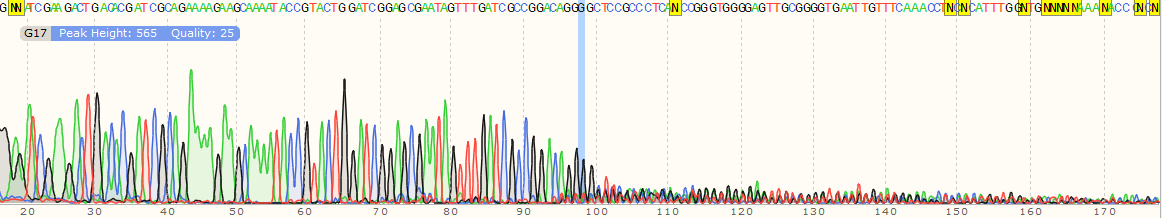


**>CTL4 sequencing (lane 4)**

CGAGACTGACACGATCGCAGAAAAGAAGCAAAATNCCGTACTGGATCGGAGGGACTAGNTAGATCGNCGGNNNGGCCAANTCCTAAACCTTTTNNAGCTCTTTTTTCCACCCCCTTTTTTNCATTTCCCAAAAAAATATCTCAATTTCACNNCACCCCAAATTGATCCCAATTCCCCCACCCCCAAAAANGTGTNTCNCTNCCCCCACCCNNNTGAGGAANTCTCNCTTGNTTNNTTACNNCCATGNNCNNCTGTNNNTNTGNTNCNTNNNAATGNNTGAGCCNGGNNTGNCGAT


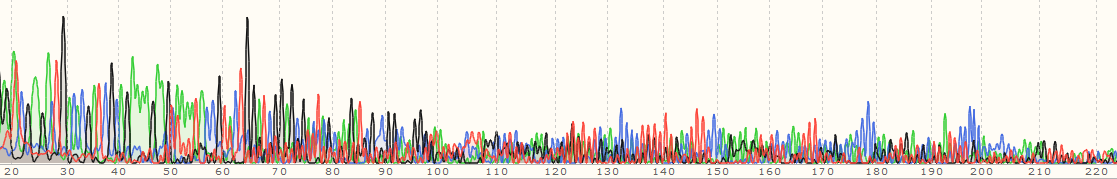


**>CTL4 sequencing (lane 6)**

CGNAGACTGACACGATCGCAGAAAAGAAGCAAAATACCGTACTGGATCGGAGCGAATAGTTTGATCGCCGGACAGGGGCTTACCACNNCGTGGTGNGNCGNGTNANTTGTTNNTCCCCCGAAACNTTCTNCNNNANATNGNANTCCACGNTNNNCACCCGGANGNNACNNAATGNCCCCCNTNNNCNGATTTTGATTNATNCNCCTTGCTCTNCAAGTACNNTNNCCTTGATTNAGGACNNCCATNTANACCCNCANNCNNTNNNNNNTGGNTCTNNACTCNAGCAAN


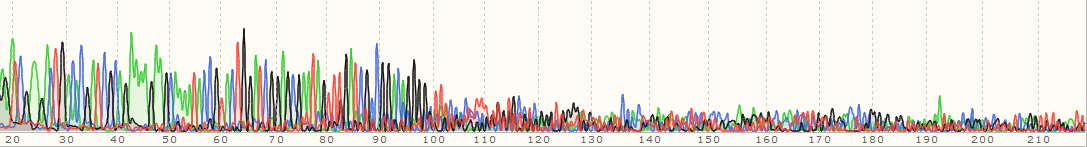

Supplement: S4 File — (DOCX) [file pbio.3001515.s011.docx]
